# Establishment of tumor-specific copy number alterations from plasma DNA of patients with cancer

**DOI:** 10.1002/ijc.28030

**Published:** 2013-01-15

**Authors:** Ellen Heitzer, Martina Auer, Eva Maria Hoffmann, Martin Pichler, Christin Gasch, Peter Ulz, Sigurd Lax, Julie Waldispuehl-Geigl, Oliver Mauermann, Sumitra Mohan, Gunda Pristauz, Carolin Lackner, Gerald Höfler, Florian Eisner, Edgar Petru, Heinz Sill, Hellmut Samonigg, Klaus Pantel, Sabine Riethdorf, Thomas Bauernhofer, Jochen B Geigl, Michael R Speicher

**Affiliations:** 1 Institute of Human Genetics, Medical University of GrazHarrachgasse 21/8, A-8010, Graz, Austria; 2 Division of Oncology, Medical University of GrazAuenbruggerplatz 15, A-8036, Graz, Austria; 3 Institute of Tumor Biology, University Medical Center Hamburg EppendorfMartinistr. 52, D-20246, Hamburg, Germany; 4 Department of Pathology, General Hospital Graz WestGoestingerstrasse 22, A-8020, Graz, Austria; 5 Department of Obstetrics and Gynecology, Medical University of GrazAuenbruggerplatz 14, A-8036, Graz, Austria; 6 Institute of Pathology, Medical University of GrazAuenbruggerplatz 25, A-8036, Graz, Austria; 7 Division of Hematology, Medical University of GrazAuenbruggerplatz 38, A-8036, Graz, Austria

**Keywords:** plasma DNA, tumor monitoring, predictive and prognostic biomarker, copy number changes

## Abstract

With the increasing number of available predictive biomarkers, clinical management of cancer is becoming increasingly reliant on the accurate serial monitoring of tumor genotypes. We tested whether tumor-specific copy number changes can be inferred from the peripheral blood of patients with cancer. To this end, we determined the plasma DNA size distribution and the fraction of mutated plasma DNA fragments with deep sequencing and an ultrasensitive mutation-detection method, *i.e.,* the Beads, Emulsion, Amplification, and Magnetics (BEAMing) assay. When analyzing the plasma DNA of 32 patients with Stage IV colorectal carcinoma, we found that a subset of the patients (34.4%) had a biphasic size distribution of plasma DNA fragments that was associated with increased circulating tumor cell numbers and elevated concentration of mutated plasma DNA fragments. In these cases, we were able to establish genome-wide tumor-specific copy number alterations directly from plasma DNA. Thus, we could analyze the current copy number status of the tumor genome, which was in some cases many years after diagnosis of the primary tumor. An unexpected finding was that not all patients with progressive metastatic disease appear to release tumor DNA into the circulation in measurable quantities. When we analyzed plasma DNA from 35 patients with metastatic breast cancer, we made similar observations suggesting that our approach may be applicable to a variety of tumor entities. This is the first description of such a biphasic distribution in a surprisingly high proportion of cancer patients which may have important implications for tumor diagnosis and monitoring.

**What's new?**

Tumors shed their DNA into the bloodstream. This DNA can be detected, but whether it's useful as a diagnostic tool hasn't been clear from existing reports. Rather than attempt to pick out specific mutations, however, this study asked whether it would be possible to get a genome-wide view of tumor-specific copy number changes from this circulating tumor cell DNA. When they analyzed the plasma DNA of patients with colorectal cancer, the authors found that about a third of the patients had plasma DNA that fell into two distinct size categories, and this correlated with higher numbers of circulating tumor cells. They could then detect tumor-specific copy-number changes from this plasma DNA. Further developing this non-invasive acquisition of tumor material could aid in tailoring specific disease treatment strategies.

Recent advances in the understanding of the molecular mechanisms of cancer have highlighted the need for personalized medicine approaches not only in terms of prognosis but also for diagnostic strategies. Extensive work has resulted in the identification of biomarkers that have been implemented in cancer clinical practice at several levels: prognostics, predictive and pharmacokinetic biomarkers. For example, in colorectal cancer (CRC), the *KRAS* mutations in Exon 2 (Codons 12 and 13) represent a paradigm that has been established as a negative predictive marker for treatment with epidermal growth factor receptor (EGFR) inhibitors such as cetuximab and panitumumab.^1^ The discovery of further biomarkers will be accelerated by the recent advances in cancer genomics.

Access to accurate and sensitive methods for the detection of such predictive biomarkers is of utmost importance to the clinical oncologist. Numerous studies have tried to identify such biomarkers in the form of circulating DNA because tumors shed DNA into the circulation that can be detected by appropriate means. However, early reports proposing that the presence or absence of circulating DNA or its concentration was of diagnostic value^2^,^3^ have been called into question.^4^–^7^ Analyses of plasma/serum DNA for loss of heterozygosity^8^,^9^ or for tumor-related methylation patterns^10^,^11^ often lack specificity. Furthermore, it has been proposed that the detection of mutations in peripheral blood, which have previously been identified in the corresponding primary tumor from the same patient, may provide a specific biomarker of disease burden. Therefore, multiple studies focused on the detection of such specific and predetermined mutations.^12^–^18^ For example, the emergence of *KRAS* mutant clones as evidence for acquired resistance to targeted EGFR blockade in patients with CRC has been inferred from the analysis of plasma DNA.^19^,^20^ A broader approach is the use of targeted amplicon sequencing for the simultaneous analysis of multiple cancer driver genes.^21^ However, the sensitive and specific detection of a mutated base in a vast excess of normal DNA requires specialized techniques that are currently beyond the scope of many diagnostic laboratories.^12^–^14^,^18^

In our study, we investigated whether instead of a targeted approach a genome-wide view of tumor-specific copy number changes can be established from peripheral blood, *i.e.,* plasma DNA from patients with cancer. To this end, we simultaneously quantified the normal and mutant DNA molecules in a given sample and established genome-wide copy number changes from plasma and compiled detailed data on copy number changes in relation to the respective primary tumors and metastases. We validated these observations with blood samples from 35 patients with breast cancer. Our results suggest that complex tumor genomes can be reconstructed from the peripheral blood of patients with cancer. Our approach may be of interest for two scenarios: first, as a research tool to investigate a tumor genome at late stage disease, which is not commonly studied. Second, it may pave the way for new disease monitoring strategies.

## Material and Methods

### Preparation of plasma DNA

Whole blood (9 ml) was collected in routine ethylenediamine tetraacetic acid (EDTA) Vacutainer tubes (BD Biosciences, Heidelberg, Germany). To stabilize cell membranes and to impede cell lysis, 0.225 ml of a 10% neutral buffered solution containing formaldehyde (4% weight per volume) (Sigma-Aldrich, Vienna, Austria) was added immediately after blood withdrawal. Blood samples were gently inverted, stored at room temperature and further processed within 2 hr. Plasma was prepared according to Ref.^22^. In brief, tubes were centrifuged at 200*g* for 10 min with the brake and acceleration powers set to zero, followed by a subsequent centrifugation step at 1600*g* for 10 min. The supernatant was collected, transferred to a new 15 ml tube and spun at 1600*g* for 10 min. The plasma was carefully aliquoted into new 2 ml Eppendorf tubes and stored at −80°C.

DNA was isolated from plasma samples using the QIAamp DNA Blood Mini Kit (Qiagen, Hilden, Germany) or the Qiagen Circulating Nucleic Acids Kit (CNA) (Qiagen, Hilden, Germany) according to the manufacturer's instructions. DNA was eluted in 30 μl of distilled water for Mini Kit extractions and in 100 µl for CNA Kit, respectively.

### Further methods

All other methods used in this article are detailed in the Supporting Information.

## Results

### A subset of patients with CRC had a biphasic plasma DNA size distribution

We analyzed the peripheral blood of 32 patients with advanced stage CRC (Supporting Information and Supporting Information Table 1). We started with measuring of the plasma DNA concentrations of healthy controls and patients with advanced-stage CRC. Compared to controls (mean: 15.21 ng/ml; median: 14.37 ng/ml; range: 12.20–19.51 ng/ml), patients showed invariably higher values with substantial variability (mean: 275.35 ng/ml; median: 139.0 ng/ml; range: 22.44–1,037.49 ng/ml) (*p*<0.0001).

**Figure 1:**
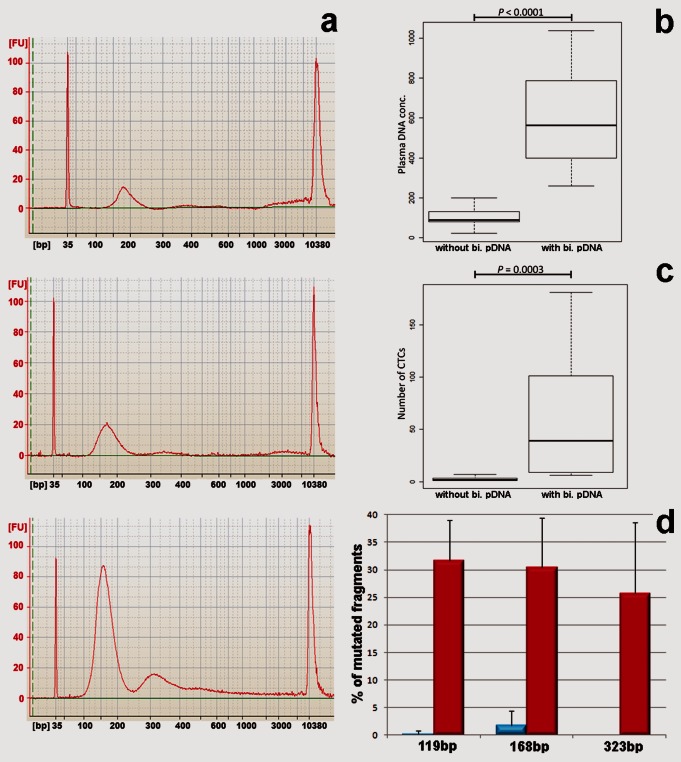
Characteristics of plasma DNA from healthy controls and patients with advanced-stage colorectal cancer (CRC). (*a*) Size distribution of plasma DNA fragments from a healthy donor (upper plot), Patient #11 (center plot) and Patient #22 (lower plot). Normalization was performed using two internal markers, visible as high, narrow amplitudes at Positions 35 and 10.380 bp, respectively. For each analysis, 800 pg of DNA was used. (*b*) Patients with a biphasic plasma DNA size distribution (with bi. pDNA) have higher plasma DNA concentrations compared to patients lacking the second peak (without bi. pDNA). (*c*) The occurrence and number of CTCs is closely correlated with a biphasic plasma DNA size distribution. (*d*) Deep sequencing using three different sequencing reaction sizes (*i.e*., 119, 168 and 323 bp) identified few mutated *KRAS* fragments in patients without a biphasic plasma DNA size distribution (blue), but high levels of mutated *KRAS* fragments in patients with a biphasic plasma DNA size distribution (red) (the errors bars represent SDs).

Because mutant DNA fragments in the blood circulation of cancer patients were reported to be degraded relative to nonmutant DNA fragments,^12^ we used a microfluidics-based platform for sizing. We observed an enrichment of plasma DNA fragments within the range of 85–230 bp in the healthy controls (Fig. 1*a*, upper plot). Plasma DNA fragments within this size range have been associated with the release of DNA from apoptotic cells after enzymatic processing.^12^,^23^ There was no significant qualitative difference regarding the size distribution of plasma DNA fragments between the healthy controls and 21 (65.6%) of the CRC cases despite the higher concentrations of plasma DNA in the latter group (Fig. 1*a*, center plot). However, in 11 (34.4%) patients, we observed a second peak consisting of DNA fragments with a size range of 240 to 400 bp, or even longer in some cases (Fig. 1*a*, lower plot). Interestingly, the patients with biphasic plasma DNA size distributions had significantly higher plasma DNA concentrations (mean: 604 ng/ml; median: 562 ng/ml; range: 260–1,037 ng/ml) than patients without a second peak (mean: 103 ng/ml; median: 89 ng/ml; range: 22–201 ng/ml) (*p*<0.0001; Fig. 1*b*).

### The biphasic plasma DNA size distribution correlates with circulating tumor cell occurrence

To investigate whether the DNA size distributions detected might reflect the contributions of different tumor cell populations releasing their DNA into the circulation around the time of blood collection, we determined the number of circulating tumor cells (CTCs). We used the FDA-approved Veridex system for CTC detection^24^ in 30 of the 32 patients (the analysis could not be performed for patients #10 and #35). We observed a clear correlation between the presence of a biphasic plasma DNA size distribution and CTC occurrence. In the patient group with a biphasic distribution (*n*=10), we found a mean number of 52 CTCs (median: 35; range: 0–181). In contrast, the mean CTC number in patients without a biphasic plasma DNA size distribution (*n*=20) was only 1.5 (median: 1; range: 0–7) (*p*=0.0003; Fig. 1*c*).

### Deep sequencing of plasma DNA for KRAS mutations

To investigate whether mutant DNA fragments were present in both the first and second peaks in the plasma DNA fragment size distribution, we used ultra-deep pyrosequencing. We used eight patient samples with and without biphasic plasma DNA size distributions and *KRAS* mutations in their corresponding primary tumors to establish the percentage of mutated plasma DNA fragments for different sequencing reaction sizes (*i.e*., 119, 168 and 323 bp). In four patients (#6, #10, #25 and #38) with biphasic plasma DNA size distributions, we found a high percentage of mutated DNA fragments compared to the other four patients without biphasic peak (#7, #11, #15 and #16) (Fig. 1*d*; Supporting Information Table 2). With the exception of Patient #25, the fraction of mutant molecules was not dependent on the size of the amplicon, suggesting that for these patients (#6, #10 and #38) DNA from the first and the second peaks consists largely of tumor DNA. By contrast, in the four patients who had only the first apoptosis-related peak deep sequencing identified mutated *KRAS* fragments at low levels in two cases (#11 and #15) and none in the other two patients (#7 and #16) (Fig. 1*d*; Supporting Information Table 2). In these four patients, mutated DNA fragments were not observed in the 323 bp sequencing reaction.

### Genome-wide estimation of copy number changes in plasma DNA

**Figure 2:**
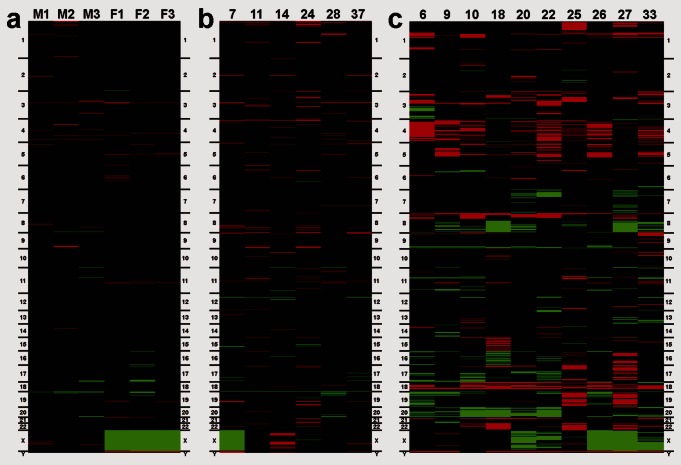
Heat maps of plasma DNA profiles from healthy controls and patients with advanced-stage colorectal cancer (CRC). (*a*) Heat map of plasma DNA profiles from healthy donors (black: balanced; red: under-represented; green: over-represented). As we used male reference DNA in all experiments, female plasma DNA samples have a relative over-representation of the X chromosome and an under-representation of the Y-chromosome (F1–F3), whereas male samples have balanced sex chromosomes (M1–M3). (*b*) Heat maps of six exemplary patients (#7, #11, #14, #24, #28 and # 37) that lack a biphasic plasma DNA size distribution. (*c*) Heat maps of plasma DNA from 10 patients (#6, #9, #10, #18, #20, #22, #25, #26, #27 and #33) that have a biphasic plasma DNA size distribution.

We postulated that, in addition to *KRAS*-mutated DNA fragments, other tumor DNA fragments should be present in the circulation of these patients and that these might provide insights about the tumor genome. To address this hypothesis, we generated random DNA libraries by converting the plasma DNA fragments into polymerase chain reaction (PCR)-amplifiable OmniPlex Library molecules flanked by universal priming sites for whole-genome amplification (WGA) and subjected the WGA products to array CGH on a 60 K microarray platform. This array platform consists of 55,077 oligonucleotides, and we calculated for each of these oligonucleotides whether the ratio values were decreased, balanced or increased. Plasma DNA from six healthy controls showed a mean of 4,026 oligonucleotides from the 55,077 oligonucleotides (7.2%; range: 3,606–4,321; 6.4%–7.7%), with aberrant ratio values for the autosomes (Fig. 2*a*) (details about these calculations are in the Supporting Information). When we performed array CGH with plasma DNA from patients who had only the first plasma DNA peak (*n*=21), we observed an increase in oligonucleotides with aberrant ratio values with a mean of 5,572 oligonucleotides (10.0%; range: 4,587–7,469; 8.2%–13.4%; *p*=0.001 compared to the healthy controls) (Fig. 2*b*). Because these oligonucleotides involved only single, nonadjacent oligonucleotides, they most likely represented artifacts of the fragmentation and/or amplification process. In contrast, the plasma DNA in 10 of the 11 patients with a second peak had a mean number of oligonucleotides with copy number changes of 12,476 (22.3%; range: 9,117–14,915; 16.3%–26.7%), which differed highly significantly from both the aforementioned CRC cases and the healthy controls (*p*<0.0001 each) (Fig. 2*c*).

### Tumor-specific, genome-wide imbalances in plasma DNA with a biphasic size distribution

Because most of the aforementioned copy number changes in the plasma DNA with biphasic size distribution involved large regions of adjacent oligonucleotides, we determined whether or not they were tumor specific by comparing them to copy number changes of the primary tumor and, if available, metastatic material. As an exemplary case, we present Patient #6 in greater detail. When the initial diagnosis was made in Patient #6, he already had metastases in the liver, bones, abdominal lymph nodes and peritoneum. At this time, only a biopsy was obtained from the primary tumor. Five months later, a cerebellar metastasis was completely resected, and 3 months after that, we collected blood for our analyses—*i.e*., 8 months after the initial diagnosis. Thus, multiple tumor sites could have released tumor DNA into the circulation at the time of blood collection.

**Figure 3:**
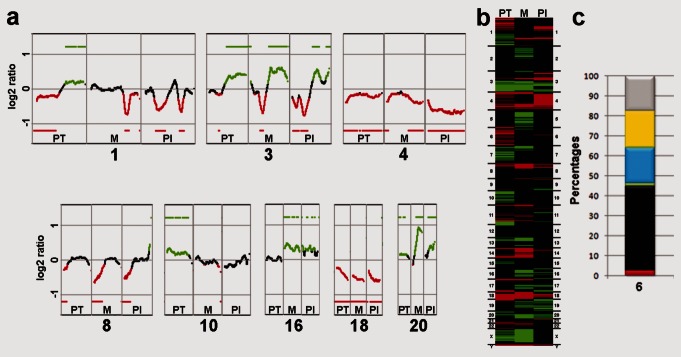
Comparison of copy number changes in the primary tumor, metastasis and plasma DNA of Patient #6. (*a*) Comparison of the ratio profiles of representative chromosomes (1, 3, 4, 8, 10, 16, 18 and 20; indicated by numbers below the copy number changes) between the primary tumor (PT, left column), metastasis (M, center column) and plasma DNA (Pl, right column). The single green and red bars summarize the regions that were gained or lost based on all iterative calculations of our algorithm (Supporting Information). The black profile regions represent balanced regions, lost regions appear in red and gained regions are shown as green (complete profiles are depicted in Supporting Information Fig. 1*a*). (*b*) Heat maps comparing the copy number changes in the primary tumor (PT), metastasis (M), and plasma DNA (Pl; Black: balanced regions; red: under-represented regions; green: over-represented regions). (*c*) The bar chart combines information on copy number changes and their occurrence in the primary tumor, metastasis and plasma DNA. It displays the percentages of chromosomal regions that were commonly lost (red), balanced (black) or gained (green) in all three samples, shared by metastasis and plasma DNA only (blue), shared by primary tumor and plasma DNA only (yellow) or unique to the plasma DNA (gray).

We noted marked copy number differences between the primary tumor and the cerebellar metastasis in Patient #6 (Supporting Information Fig. 1, Panels 1 and 2), indicative of the presence of several malignant cell clones. We found numerous copy number changes in the plasma DNA (Supporting Information Fig. 1, Panel 3) and performed a detailed analysis consisting of the following steps: First, we compared the ratio of the profiles of the available material, *i.e.,* primary colorectal tumor, cerebellar metastasis and plasma DNA (Fig. 3*a*). We then constructed detailed heat maps showing whether the copy number status was decreased, balanced or increased for each oligonucleotide on our array platform (Fig. 3*b*). Finally, we determined whether the copy number status for each oligonucleotide occurred only in the plasma DNA or also in the primary tumor and/or metastasis (Fig. 3*c*). These analyses revealed that the copy number status of 46.3% of the oligonucleotides on our array platform was present in all three lesions; 18.1% was shared by the primary tumor and plasma DNA, 18.0% was shared by the metastasis and plasma DNA and 17.6% was unique to the plasma DNA (Fig. 3*c*). This suggested that the observed changes in the plasma DNA were tumor-specific and were largely caused by clones both from the primary and the cerebellar metastatic tumor. As the cerebellar metastasis had been removed, cells from this metastatic clone are apparently still present in the patient. Whether the copy number changes observed only in the plasma DNA could have resulted from the other metastases or from parts of the primary tumor that were not included in our analysis remains unknown.

In addition to Patient #6, we had three other patients (*i.e.,* #9: Supporting Information Fig. 2; #26: Supporting Information Fig. 3; #33: Supporting Information Fig. 4) for whom material from the primary tumor and metastases were available for comparison. Altogether, the plasma DNA samples from these four patients (*i.e*., #6, #9, #26 and #33) displayed an identical copy number status (*i.e.,* lost, balanced and gained) in all three samples (primary, metastasis and plasma DNA) for an average of 54.7% (median: 54.3%; range: 46.3%–63.6%) of all oligonucleotides. About 10% (median: 9.4%; range: 3.1%–18.1%) of plasma DNA copy number changes were unique to the metastasis and, *vice versa*, ∼14.5% (median: 16.2%; range: 2.9%–22.7%) were only present in the primary tumor. About 20.9% (median: 18.7%; range: 15.7%–30.4%) of plasma DNA changes were observed only in the plasma DNA but not in the primary tumor and metastasis. However, all four patients had additional metastases at various sites (Supporting Information Table 1), which were not accessible to us. Thus, copy number changes observed only in the plasma DNA could reflect alterations from a metastatic site not included in our analysis.

For another patient (#27), we had material from the primary tumor only, and we obtained comparable results with the plasma DNA, *i.e.,* copy number changes similar to those observed in the primary tumor (Supporting Information Fig. 5). By contrast, we did not find copy number changes of large contiguous chromosomal regions in the plasma DNA of Patient #38 that unequivocally correspond to those of the primary tumor or metastases (Supporting Information Fig. 6).

### Plasma DNA analysis in the absence of material from the primary tumor or metastasis

In three cases, *i.e.,* #10 (Supporting Information Fig. 7*a*), #20 (Supporting Information Fig. 7*b*) and #25 (Supporting Information Fig. 7*c*), no further tumor material was available for analyses. However, in each of these cases, we were able to establish array-CGH profiles reminiscent of copy number changes frequently observed in colon cancer (http://www.progenetix.net), such as loss on 8p and gains on 8q and 20.

**Figure 4:**
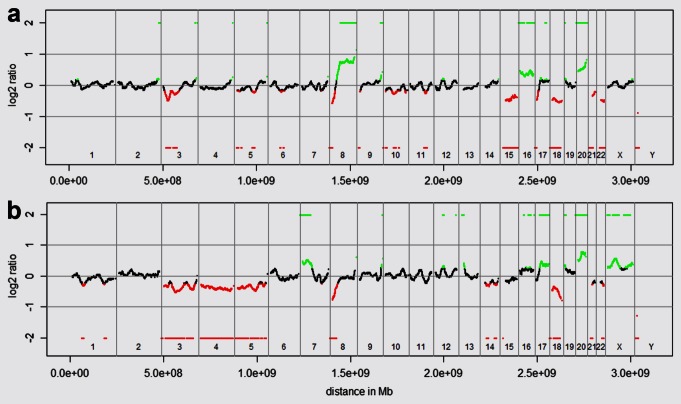
Array-CGH from two cases where only small biopsies had been taken at the time of diagnosis, so insufficient material was available for further analyses. (*a*) The plasma DNA from Patient #18 revealed typical CRC-related copy number changes, such as loss on 8p and gains on 8q and 20. (*b*) Plasma DNA ratio profile from Patient #22 demonstrating losses on chromosomes 3, 4, 5, 8p and 18 and gains on chromosomes 7p, 17q and 20.

In two cases (*i.e.,* #18, #22), insufficient material was available because only small biopsies had been taken at the time of diagnosis. In Patient #18, our plasma DNA analysis again revealed typical CRC-related copy number changes, such as loss on 8p and gains on 8q and 20 (Fig. 4*a*), whereas in Patient #22, our plasma DNA analysis revealed losses on chromosomes 3, 4, 5, 8p and 18 and gains on chromosomes 7p, 17q and 20 (Fig. 4*b*). These results suggest that tumor-specific copy number profiles can be established even in cases where for various reasons no material from the primary tumor or metastasis is available.

### BEAMing analysis

As all patients included in our study had metastatic disease showing disease progression at the time of blood collection, the significant variability of plasma DNA size distribution and concentrations as well as CTC number was unexpected. Furthermore, we were puzzled that even deep sequencing did not find evidence for overt mutant DNA fragments in some of our patients. Therefore, we tested patients for the presence of *KRAS* mutations with a more sensitive approach, *i.e.,* BEAMing, which has the capacity to detect and enumerate mutant and wild-type DNA when present at ratios greater than 1:10,000^12^,^25^ (http://www.inostics.com). To exclude any variation from sample collection and handling, we performed serial analyses with blood samples from two patients (L1 and L2) collected on five (L1) or four (L2) consecutive days. These patients had end-stage, highly metastatic colon cancer and received only palliative treatment (no chemotherapy or radiation therapy), which should have no impact on the tumor burden. In both patients the *KRAS* G12V mutation had been detected in the respective primary tumors.

**Figure 5:**
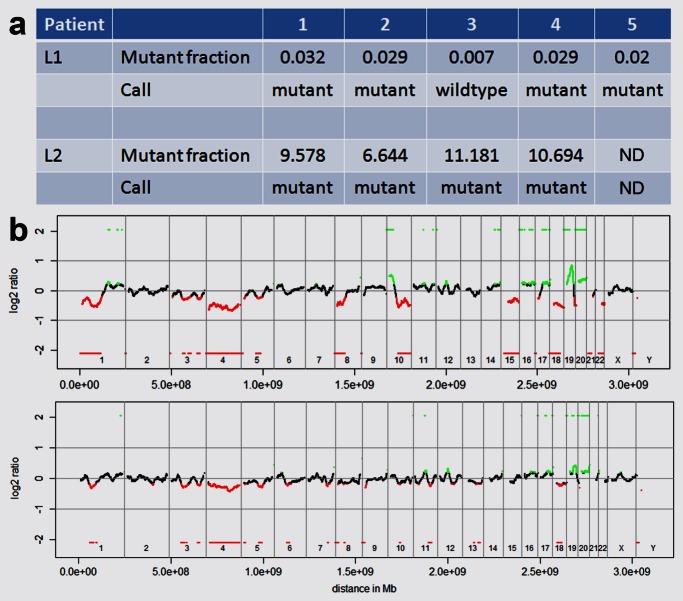
Summary of the BEAMing analysis and representative array-CGH profiles from one representative patient. (*a*) Mutant fraction and call (*i.e*., either mutant or wildtype) as established by BEAMing for two patients. (*b*) The upper panel illustrates the array-CGH profile of the primary tumor, the lower panel the corresponding profile established with plasma DNA from Day 3.

In Patient L1, the respective mutant fractions were close to the BEAMing detection limit, given as 0.02, on Days 1, 2, 4 and 5 or even below the detection limit on Day 3 (Fig. 5*a*). Accordingly, we observed invariably balanced array-CGH profiles on each day. This suggests that even ultrasensitive methods may be incapable of detecting mutant DNA fragments in some patients with highly metastatic disease.

In contrast, in Patient L2, the mutant fractions were between 6.644 (Day 2) and 11.181 (Day 3) (Fig. 5*a*). On Day 3, there was a biphasic plasma DNA size distribution, and we observed in the array-CGH analyses copy number changes on chromosomes 3, 4, 18, 19 and 20, which we had previously also observed in the primary tumor (Fig. 5*b*). Although the plasma DNA size distribution was monophasic on the other days, some of these copy number changes were also visible in the array-CGH profiles on these days.

### Correlation with clinical parameters

Although this was a pilot study, we then attempted to correlate our findings on plasma DNA size distribution and CTC number with clinical parameters. We did not find any correlation between the levels of the established CRC tumor markers CEA (Supporting Information Fig. 8*a*) and CA19-9 (Supporting Information Fig. 8*b*) and plasma DNA concentration or CTC numbers. The percentage of patients with a biphasic plasma DNA size distribution was 67%, 44% and 38% for patients with metastases in bone (*n*=6), liver (*n*=25) and peritoneal carcinomatosis (*n*=8), respectively, and only 9% for patients with lung metastasis (*n*=11) (Supporting Information; Supporting Information Table 3). These percentages were almost identical for patients with more than six CTCs, *i.e*., 67%, 36%, 9% and 38%, for patients with metastasis in bone, liver, lung and peritoneal carcinomatosis, respectively. In fact, only one of the 11 patients with lung metastasis (Patient #38) demonstrated both a biphasic plasma DNA size distribution and a high CTC number, according to the Veridex system.

### Analyses of plasma DNA from patients with breast cancer

**Figure 6:**
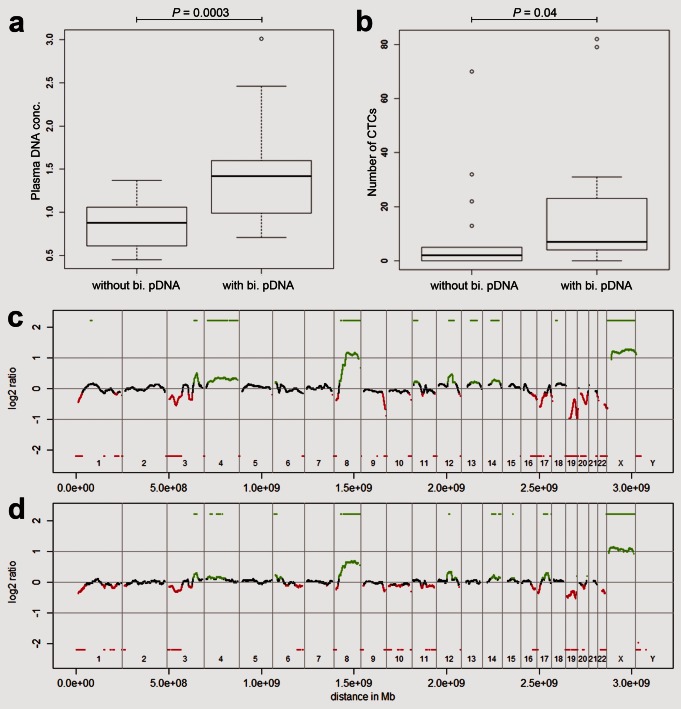
Evaluation of blood from 35 patients with breast cancer and representative array-CGH profiles. (*a*) Breast cancer patients with a biphasic plasma DNA size distribution (with bi. pDNA) have increased plasma DNA concentrations (here depicted as log10 ratio) compared to patients lacking the second peak (without bi. pDNA). (*b*) The number of CTCs is also increased in patients with a biphasic plasma DNA size distribution. (*c*) Array CGH profile of the primary tumor of breast cancer Patient #20. (*d*) Corresponding array-CGH profile obtained with plasma DNA from Patient #20, who had a biphasic plasma DNA size distribution.

To test whether our observations can be extended to other tumor entities, we collected blood from 35 patients with breast cancer. Indeed, we observed the biphasic plasma DNA size distribution in 14 (40%) patients, which again correlated with the plasma DNA concentration (Fig. 6*a*) and number of CTCs (Fig. 6*b*). In these cases, we could again reconstruct tumor specific copy number changes from the plasma DNA (Figs. 6*c*–6*d*).

## Discussion

A major goal of cancer medicine is to move from fixed treatment regimens to therapies tailored to a patient's individual tumor. Following recent breakthroughs in genomics technology, major efforts to identify potentially informative mutations using next-generation sequencing are under way.^26^ However, such detailed analyses are usually performed with samples obtained during the initial diagnosis and/or metastasis.^27^,^28^ All these genomic studies have confirmed that tumor genomes are complex and highly prone to changes. In our study, we addressed whether complex tumor genomes may be inferred noninvasively from the peripheral blood of patients with cancer.

We observed that the plasma DNA and CTCs varied significantly in our cohort, although all patients included in our study had metastatic disease showing disease progression at the time of blood collection. Some cancer patients with only the first peak have higher plasma DNA levels compared to healthy controls but, as confirmed by deep sequencing, BEAMing and array-CGH, a very low percentage of mutated DNA fragments. This is consistent with necrotic neoplastic cells being engulfed by macrophages, which involves the killing of neoplastic cells and the surrounding stromal and inflammatory cells.^12^ The released DNA contains multiple wild-type DNA sequences, which may explain the increase in total, nonmutant circulating DNA.

In contrast, a biphasic plasma DNA size distribution may indicate a distinct biological process, because its occurrence is associated with very high plasma DNA levels, elevated percentages of mutated DNA fragments in the circulation, and an increased number of CTCs. The biphasic plasma DNA distribution likely reflects massive cell destruction with direct shedding of DNA from tumor cells and cellular fragments into the bloodstream. As the number of CTCs identified by the Veridex system is relatively small, CTCs by themselves likely contribute only small amounts of tumor DNA into the circulation. Hence, the biphasic plasma DNA size distribution is more likely due to various mechanism of release at the site of the tumor, for example, when the tumor has invaded through blood vessels. The first peak consisting of plasma DNA fragments within the range of 85–230 bp could be released from apoptotic cells after enzymatic processing. Previously plasma DNA fragments within this size range had been associated with the release of DNA from apoptotic cells after enzymatic processing, because the length of these fragments corresponds approximately to the DNA wrapped around a nucleosome (∼142 bp) plus a linker fragment (∼20 bp).^12^,^23^ Accordingly, the DNA fragments from the second peak likely represent di- and trinucleosomal DNA fragments. Hence, the co-occurrence of larger DNA fragments suggests that their broken nuclear products were subject to shorter degradation times in the circulation. Alternatively, the biphasic DNA may be a consequence of saturation of DNA degradation mechanisms in the plasma due to high amounts of DNA. In contrast, we did not observe significant amounts of DNA fragments from necrotic cells, which were associated with DNA fragment sizes larger than ∼10,000 bp.^29^ Thus, our results may explain previously reported differences in the detection rates of abnormal forms or quantities of DNA in plasma^5^–^7^,^30^ and why most studies found that plasma tumor DNA fragments are degraded,^8^,^12^,^13^,^21^ whereas only few reported an increased integrity.^31^,^32^ At present, we do not know whether the biphasic plasma DNA size distribution represents a specific tumor signature, as we have not yet studied other conditions in which plasma DNA may be increased, such as chronic inflammatory diseases.

Our genome wide analyses contrast with numerous previous studies that have attempted to identify abnormal forms or quantities of DNA in plasma or serum.^4^,^6^,^7^ However, these previous approaches had usually only monitored a single target from plasma DNA,^12^,^15^,^16^ we have developed a novel technology to establish genome-wide tumor-specific copy number changes from plasma DNA with easy means. This allows novel diagnostic strategies in cases where sufficient material from the primary tumor is not available for further analyses, and it enlarges options for disease monitoring. Very recently, Chan *et al*.^33^ analyzed plasma DNA from four patients with hepatocellular carcinoma and one patient with synchronous breast and ovarian cancer by shotgun massively parallel sequencing to identify tumor-associated copy number changes. Another study, which was published while our study was in the review process, also used a whole-genome sequencing approach for the analysis of plasma of 10 colorectal and breast cancer patients.^34^ Their results suggest that next-generation sequencing based approaches may increase the resolution of copy number analyses from circulating DNA.

However, our study differs from previous plasma DNA related articles in one important issue. Most studies reported that plasma DNA reflects well the clinical course of a disease and that the amount of mutated DNA fragments would correlate with the tumor burden.^13^,^14^,^19^–^21^,^33^ In contrast to these reports, a surprising finding was that some of our patients with progressive, metastatic disease seem to release no mutant DNA fragments in measurable quantities. This was especially exemplified in Patient L1, who had highly metastatic, progressive end-stage disease where even an ultrasensitive approach such as BEAMing was not capable of detecting mutated DNA fragments. In addition, we had in our cohort of metastatic patients with progressive disease several other patients in whom deep sequencing did not detect mutated DNA fragments. If a close correlation between tumor burden and tumor-specific DNA fragments in the circulation would exist, we should have seen in all of our patients tremendous amounts of these fragments in their blood. A possible explanation may be that due to the short half-life of plasma DNA, which had been estimated to be merely 16 min,^35^ in some of our cases no significant amounts of tumor DNA had been released into the circulation several hours before our blood collection. Hence, testing of the size distribution of plasma DNA fragments may be an easy screening tool to identify blood samples, which are worth more detailed plasma DNA and/or CTC analysis.

The next challenge will be to test such approaches for the serial monitoring of cancer patients and to evaluate their potential for the early-stage analysis of peripheral blood. However, our study suggests that noninvasive access to tumor material immediately before specific anti-tumor treatment initiation in advanced cases may become possible for the re-evaluation of the cancer genome and possibly the tailored management of treatment choices.

## Supplementary material

**Supplementary Table 1.** Summary of clinical characteristics of the 32 colorectal carcinoma (CRC) patients.

**Supplementary Table 2.** Ultra-deep pyrosequencing with sequencing reaction sizes of 119 bp, 168 bp, and 323 bp in patients without (#7, #11, #15, and #16), and with biphasic plasma-DNA distribution (#6, #10, #25, #38). For each sample, the top line indicates the percentage of mutated DNA fragments (column “% mutated”) and the number of reads (column “reads”), whereas the bottom line indicates the same after whole-genome amplification (WGA). The column labeled “Mutation” lists the *KRAS* mutation found in the respective primary tumor.

**Supplementary Table 3.** Sites of metastases and their association with the biphasic plasma DNA (bi-pDNA) distribution and number of CTCs (NA: not available).

**Supplementary Table 4.** Mapping of regions involved in artifacts after whole-genome amplification (WGA) of severely fragmented plasma-DNA. The first column lists the chromosomal region, the second and third columns the exact borders of the respective region, the fourth column the size of the region (in bp), the fifth column the number of oligos on the array-platform involved in the artifact and the column labeled “in/de” whether the artifact resulted in increased or decreased ratio values.

**Supplementary Figure 1** Evaluation of available material for patient #6. For all array CGH profiles the multicolor bar codes at the top or bottom of the ratio profiles illustrate the results obtained during the iterative calculations with various window sizes, the single green and red bars summarize the regions which were gained or lost based on all calculations (Methods). Black parts in the profile represent balanced regions, lost regions appear in red and gained regions in green. The respective heat maps show the copy number status (red: under-represented; black: balanced; green: over-represented). The bar charts display the percentages of chromosomal regions, which were commonly lost (red), balanced (black), or gained (green) in all analyzed samples; shared by metastasis and plasma DNA only (blue); shared by primary tumor and plasma DNA only (yellow); or unique to the plasma DNA (gray). (a) Array CGH profiles of the primary tumor (panel 1), metastasis (panel 2), and plasma DNA (panel 3) for patient #6.

**Supplementary Figure 2** Analysis of material from patient #9. Array CGH profiles of the primary tumor (panel 1), metastasis (peritoneal carcinomatosis; panel 2), plasma DNA (panel 3), heat map (panel 4), and bar chart (panel 5). For explanations regarding the color codes see legend of Supplemetary Figure 1.

**Supplementary Figure 3** Analysis of material from patient #26. Array CGH profiles of the primary tumor (panel 1), liver metastasis (panel 2), plasma DNA (panel 3), heat map (panel 4), and bar chart (panel 5). For explanations regarding the color codes see legend of Supplemetary Figure 1.

**Supplementary Figure 4** Analysis of material from patient #33. Array CGH profiles of the primary tumor (panel 1), metastasis (liver; panel 2), plasma DNA (panel 3), heat map (panel 4), and bar chart (panel 5). For explanations regarding the color codes see legend of Supplemetary Figure 1.

**Supplementary Figure 5** Analysis of material from patient #27. Analysis of primary tumor (panel 1), plasma DNA (panel 2), and heat map (panel 3). The bar chart (panel 4) indicates percentages of commonly lost (red), gained (green), and balanced (black) oligonucleotides. Gray indicates copy number changes observed only in the plasma DNA, but not in the primary tumor.

**Supplementary Figure 6** Analysis of material from patient #38. Array CGH profiles of the primary tumor (panel 1), liver metastasis (panel 2), and plasma DNA (panel 3).

**Supplementary Figure 7** Plasma DNA profiles from three patients for which no further tumor material was available for analysis, i.e. patient #10 (a); patient #20 (b); patient #25 (c).

**Supplementary Figure 8** Correlation with tumor markers: (a) CEA (slope of the linear regression line: 0.0601; correlation coefficient: 0.27). (b) CA19-9 (graph without patient 6, whose CA19-9 value was exceptional high with 370,448; Slope of the linear regression line without patient 6: 0.0308; correlation coefficient: 0.315; with patient 6: slope: 0.0018; correlation coefficient: 0.394).

**Supplementary Figure 9** Array CGH analysis after whole genome amplification products from plasma DNA. (a) Plasma DNA analysis from female healthy donor F3 showing artifacts as listed in Supplementary Table 6 before (upper panel) and after correction (lower panel). Note that the overrepresentation of the X-chromosome (male reference DNA) is clearly visible because male reference DNA was used. (b) As in (a), but with plasma DNA from male healthy donor M2 before (upper panel) and after (lower panel) artifact correction.

**Supplementary Figure 9** Array CGH analysis after whole genome amplification products from plasma DNA. (a) Plasma DNA analysis from female healthy donor F3 showing artifacts as listed in Supplementary Table 6 before (upper panel) and after correction (lower panel). Note that the overrepresentation of the X-chromosome (male reference DNA) is clearly visible because male reference DNA was used. (b) As in (a), but with plasma DNA from male healthy donor M2 before (upper panel) and after (lower panel) artifact correction.

## References

[1] Walther A, Johnstone E, Swanton C (2009). Genetic prognostic and predictive markers in colorectal cancer. Nat Rev Cancer.

[2] Leon SA, Shapiro B, Sklaroff DM (1977). Free DNA in the serum of cancer patients and the effect of therapy. Cancer Res.

[3] Stroun M, Anker P, Maurice P (1989). Neoplastic characteristics of the DNA found in the plasma of cancer patients. Oncology.

[4] Pinzani P, Salvianti F, Zaccara S (2010). Circulating cell-free DNA in plasma of melanoma patients: qualitative and quantitative considerations. Clin Chim Acta.

[5] Schwarzenbach H, Hoon DS, Pantel K (2011). Cell-free nucleic acids as biomarkers in cancer patients. Nat Rev Cancer.

[6] Sidransky D (2002). Emerging molecular markers of cancer. Nat Rev Cancer.

[7] van der Vaart M, Pretorius PJ (2010). Is the role of circulating DNA as a biomarker of cancer being prematurely overrated?. Clin Biochem.

[8] Muller I, Beeger C, Alix-Panabieres C (2008). Identification of loss of heterozygosity on circulating free DNA in peripheral blood of prostate cancer patients: potential and technical improvements. Clin Chem.

[9] Schwarzenbach H, Alix-Panabieres C, Muller I (2009). Cell-free tumor DNA in blood plasma as a marker for circulating tumor cells in prostate cancer. Clin Cancer Res.

[10] Begum S, Brait M, Dasgupta S (2011). An epigenetic marker panel for detection of lung cancer using cell-free serum DNA. Clin Cancer Res.

[11] Gormally E, Caboux E, Vineis P (2007). Circulating free DNA in plasma or serum as biomarker of carcinogenesis: practical aspects and biological significance. Mutat Res.

[12] Diehl F, Li M, Dressman D (2005). Detection and quantification of mutations in the plasma of patients with colorectal tumors. Proc Natl Acad Sci USA.

[13] Diehl F, Schmidt K, Choti MA (2008). Circulating mutant DNA to assess tumor dynamics. Nat Med.

[14] Diehl F, Schmidt K, Durkee KH (2008). Analysis of mutations in DNA isolated from plasma and stool of colorectal cancer patients. Gastroenterology.

[15] Leary RJ, Kinde I, Diehl F (2010). Development of personalized tumor biomarkers using massively parallel sequencing. Sci Transl Med.

[16] McBride DJ, Orpana AK, Sotiriou C (2010). Use of cancer-specific genomic rearrangements to quantify disease burden in plasma from patients with solid tumors. Gene Chromosome Cancer.

[17] Nawroz H, Koch W, Anker P (1996). Microsatellite alterations in serum DNA of head and neck cancer patients. Nat Med.

[18] Yung TK, Chan KC, Mok TS (2009). Single-molecule detection of epidermal growth factor receptor mutations in plasma by microfluidics digital PCR in non-small cell lung cancer patients. Clin Cancer Res.

[19] Misale S, Yaeger R, Hobor S (2012). Emergence of KRAS mutations and acquired resistance to anti-EGFR therapy in colorectal cancer. Nature.

[20] Diaz LA, Williams RT, Wu J (2012). The molecular evolution of acquired resistance to targeted EGFR blockade in colorectal cancers. Nature.

[21] Forshew T, Murtaza M, Parkinson C (2012). Noninvasive identification and monitoring of cancer mutations by targeted deep sequencing of plasma DNA. Sci Transl Med.

[22] Dhallan R, Au WC, Mattagajasingh S (2004). Methods to increase the percentage of free fetal DNA recovered from the maternal circulation. JAMA.

[23] Lo YM, Chan KC, Sun H (2010). Maternal plasma DNA sequencing reveals the genome-wide genetic and mutational profile of the fetus. Sci Transl Med.

[24] Riethdorf S, Fritsche H, Muller V (2007). Detection of circulating tumor cells in peripheral blood of patients with metastatic breast cancer: a validation study of the CellSearch system. Clin Cancer Res.

[25] Diehl F, Li M, He Y (2006). BEAMing: single-molecule PCR on microparticles in water-in-oil emulsions. Nat Methods.

[26] Roychowdhury S, Iyer MK, Robinson DR (2011). Personalized oncology through integrative high-throughput sequencing: a pilot study. Sci Transl Med.

[27] Bignell GR, Greenman CD, Davies H (2010). Signatures of mutation and selection in the cancer genome. Nature.

[28] Kan Z, Jaiswal BS, Stinson J (2010). Diverse somatic mutation patterns and pathway alterations in human cancers. Nature.

[29] Jahr S, Hentze H, Englisch S (2001). DNA fragments in the blood plasma of cancer patients: quantitations and evidence for their origin from apoptotic and necrotic cells. Cancer Res.

[30] Pinzani P, Salvianti F, Pazzagli M (2010). Circulating nucleic acids in cancer and pregnancy. Methods.

[31] Umetani N, Kim J, Hiramatsu S (2006). Increased integrity of free circulating DNA in sera of patients with colorectal or periampullary cancer: direct quantitative PCR for ALU repeats. Clin Chem.

[32] Wang BG, Huang HY, Chen YC (2003). Increased plasma DNA integrity in cancer patients. Cancer Res.

[33] Chan KC, Jiang P, Zheng YW (2013). Cancer genome scanning in plasma: detection of tumor-associated copy number aberrations, single-nucleotide variants, and tumoral heterogeneity by massively parallel sequencing. Clin Chem.

[34] Leary RJ, Sausen M, Kinde I (2012). Detection of chromosomal alterations in the circulation of cancer patients with whole-genome sequencing. Sci Transl Med.

[35] Lo YM, Zhang J, Leung TN (1999). Rapid clearance of fetal DNA from maternal plasma. Am J Hum Genet.

