# Supplementary material for: Establishment of tumor-specific copy number alterations from plasma DNA of patients with cancer
Source: Int J Cancer. 2013 Jan 15;133(2):346–56. doi: 10.1002/ijc.28030 (PMC3708119; doi:10.1002/ijc.28030)
Supplement: Supplementary file 1 [file ijc0133-0346-SD1.doc]

**Supplementary Table 1.**

Summary of clinical characteristics of the 32 colorectal carcinoma (CRC) patients.

| **#** | **sex** | **age (years)** | **Site primary** | **Diagnosis of primary tumor = Dukes' stage (T stage, N stage (no. Positive Lnn/no. Examined Lnn), M stage, grade)*** | **Diameter of primary lesion (cm)** | ***KRAS* (primary tumor)** | **Interval between diagnosis of primary and first metastasis (months)** | **Sites of metastases** | **Largest diameter of metastasis(cm)** | **Interval between diagnosis of primary and blood collection (months)** | **Last therapy** | **Interval between blood collection and last therapy (days)** | **Dukes' stage**** | **CEA** (ref.:0-5)** | **CA-19-9** (ref.: 0-37)** |
| --- | --- | --- | --- | --- | --- | --- | --- | --- | --- | --- | --- | --- | --- | --- | --- |
| 1 | M | 69 | cecum | C (pT2 N2 (5/26) M0, G3) | 6.4 | G12V | ∅ | none | ∅ | 2 | 2 cycles FOLFOX | 12 | C | 3.4 | 21.40 |
| 2 | F | 48 | colon sigmoideum | D (pT3 N2 (7/20), M1, G3) | 6 | ND | 0 | Liver | 2 | 1 | Surgery, no chemotherapy | 45 | D | 2.5 | 8.30 |
| 3 | M | 50 | rectum | C (pT3c N1(3/25) M0, G2) | 4.5 | WT | 8 | Liver | 2.8 | 9 | 11 cycles FOLFOX | 16 | D | 7.5 | <2 |
| 6 | M | 60 | cecum | D (pT3 N1(1/6), M1, G3) | NA | G12V | 0 | Liver, abdominal lymphnodes, peritoneal carcinomatosis, bone, brain | 6 | 8 | 6 cycles Oxaliplatin | 48 | D | 4315 | 370448.00 |
| 7 | F | 45 | rectum | C (pT3 N1(3/20), M0, G3) | 3 | G13D | 14 | Lung | 2 | 28 | 12 cycles FOLFOX Folfiri | 313 | D | 16.7 | 74.50 |
| 9 | M | 70 | rectum | D (only biopsy, therefore no data about T, N, G stage available), M1 | NA | WT | 0 | Liver, lymphnodes, peritoneal carcinomatosis, bone | 1.5 | 1 | Surgery, no chemotherapy | 34 | D | 39.4 | 2312.00 |
| 10 | M | 76 | colon transversum | D (only biopsy, therefore no data about T, N, G stage available), M1 |  | G12D | 36 | Liver | 9.2 | 47 | 13 cycles FOLFOX | 14 | D | 1935 | 7517.70 |
| 11 | M | 76 | rectum | C (pT3 N2(10/22) M0, G1) | 4.7 | G12V | 13 | Liver, lung | 3.5 | 63 | 8 cycles CPT11 | 99 | D | 4.2 | 25.40 |
| 12 | M | 75 | cecum | D (pT4 N2(27/37)M1, G2) | 8.5 | G12A | 36 | Lung, abdominal lymphnodes | 5.4 | 47 | 16 cycles Folfiri | 95 | D | 3.4 | 16.90 |
| 14 | M | 69 | colon sigmoideum | C (pT3 N0 (0/29), M0, G2) | 4.5 | ND | 13 | Lung, thoracal lymphnodes | 3.2 | 20 | 6 cycles FOLFOX | 89 | D | 8.9 | 30.60 |
| 15 | F | 74 | cecum | D (pT3, N1(1/14),M1, G1) | 4.5 | G12D | 1 | Liver, lung | 5 | 16 | 6 cycles Folfiri | 22 | D | 106.4 | 279.80 |
| 16 | M | 71 | colon descendens | D (pT3bN2(40/41)MX) | 1.3 | G12D | 13 | abdominal lymphnodes | NA | 16 | 4 cycles Xeloda | 15 | D | 5 | 50.80 |
| 17 | F | 72 | colon sigmoideum | D (pT4, N1 (3/5), M1, G2) | NA | WT | 0 | Liver, lung, lymphnodes, peritoneal carcinomatosis | 3.5 | 39 | 7 cycles FOLFOX | 403 | D | 87.1 | 99.10 |
| 18 | M | 54 | recto-sigmoideum | D (only biopsy, therefore no data about T, N, G stage available), M1 | NA | WT | 0 | Liver | 5.9 | 1 | ∅ | 0 | D | 856.6 | 33.50 |
| 19 | M | 67 | colon sigmoideum | D (pT3 N2(5/11), M1, G2) | 7 | G13D | 0 | Liver | 1.2 | 15 | 12 cycles FOLFOX | 216 | D | 2.2 | <2 |
| 20 | M | 81 | flexura coli sinistra | D (only biopsy, therefore no data about T, N, G stage available), M1 | NA | WT | 0 | Liver | 7 | 14 | 12 cycles CPT11 | 28 | D | 194.9 | 9192.70 |
| 21 | M | 76 | rectum | C (pT3 N1(2/13), M0, G2) | NA | G12V | 10 | Liver, lung, bone | 2 | 61 | 4 cycles Xeloda | 688 | D | 17.4 | 20.30 |
| 22 | M | 62 | flexura coli sinistra | D (pT3 N2 (7/13) M1, G2) | 0.3 | WT | 0 | Liver, peritoneal carcinomatosis, adrenal gland | 14.5 | 2 | Surgery, no chemotherapy | 2 | D | 1.6 | <2 |
| 23 | M | 71 | rectum | D (only biopsy, therefore no data about T, N, G stage available), M1 | NA | WT | 0 | Liver, primary tumor in situ | 10.6 | 1 | ∅ | 0 | D | 10.5 | 26.20 |
| 24 | M | 69 | colon transversum | D (pT3 N1 (2/17) M1, G2) | 5.5 | WT | 18 | Liver, peritoneal carcinomatosis | 3.3 | 41 | 10 cycles Folfiri/ Avastin | 185 | D | 2.9 | 11.30 |
| 25 | M | 74 | colon sigmoideum | D (only biopsy, therefore no data about T, N, G stage available), M1 | NA | G12D | 0 | Liver, primary tumor in situ | 6 | 8 | 7 cycles FOLFOX | 67 | D | 5154.2 | 11881.20 |
| 26 | F | 70 | colon transversum | C (pT3 N1 (2/40)M0, G2) | 2.3 | WT | 31 | Liver, bone, spleen | 3.5 | 34 | 12 cycles CPT11/ Erbitux | 34 | D | 95.4 | 64.70 |
| 27 | F | 66 | sigma | D (pT4 N1(3/20), M1, G2) | 4.5 | WT | 0 | Liver | 3.5 | 29 | 9 cycles Folfiri | 110 | D | 2439.2 | 162.10 |
| 28 | M | 72 | rectum | C (pT3 N0(0/19), M0, G2) | 3.1 | WT | 134 | Liver, lung, adrenal gland | 2.5 | 151 | 10 cycles CPT11 | 138 | D | 227.6 | 168.70 |
| 29 | F | 76 | rectum | C (pT3 N0(0/21), M0, G2) | 4.3 | WT | 11 | Liver | 2.2 | 38 | 10 cycles FOLFOX | 21 | D | 38.8 | 9.70 |
| 30 | M | 71 | colon ascendens | C (pT3 N1(2/20) M0, G2) | 4 | WT | 7 | Liver | 2.6 | 27 | 5 cycles FOLFOX | 20 | D | 3689.5 | 18.70 |
| 32 | F | 67 |  | D (pT4 N2 (5/16) M1, G2) |  |  | 0 | Peritoneal carcinomatosis |  | 10 | 8 cycles FOLFOX | 69 | D | 5.1 | 70.10 |
| 33 | F | 75 | colon ascendens | C (pT4 N0 (0/12) M1, G2) | 4 | WT | 0 | Liver, peritoneal carcinomatosis, abdominal wall metastasis | 9.2 | 9 | 8 cycles Xeloda | 43 | D | 1303.3 | 4.10 |
| 34 | M | 77 | rectum | C (pT3 N2 (5/8) M0, G2) | 3 | G12D | 35 | Liver, lung | 7 | 62 | 3 cycles UFT (07/2010-10/2010) | 121 | D | 1075.1 | 456.50 |
| 35 | M | 78 | sigma | D (pT4 N1 (3/10) M1, G2) | 5 | WT | 0 | Lung, bone, lymph nodes | 1.5 | 94 | 4 cycles Folfire (10/2010-12/2010) | 104 | D | 1.1 | 11.90 |
| 37 | M | 67 | sigma | D (pT4, N1 (2/15), M1, G2) | 3 | G13D | 61 | Peritoneal carcinomatosis |  | 74 | 5 cycles Avastin/Folfiri | 277 | D | 10.2 | 208.10 |
| 38 | M | 63 | colon ascendens | B (pT3, N0 (0/28), M0, G2) | 5 | G12D | 44 | Liver, lung, bone, lymph nodes | 9 | 45 | Surgery, no chemotherapy | 0 | D | 2.6 | 19.20 |

*: at time of diagnosis; **: at time of blood collection

M: male

F: female

NA: not available

WT: wild type

y: years m: months d: days

ND: not done
