# Supplementary material for: Establishment of tumor-specific copy number alterations from plasma DNA of patients with cancer
Source: Int J Cancer. 2013 Jan 15;133(2):346–56. doi: 10.1002/ijc.28030 (PMC3708119; doi:10.1002/ijc.28030)
Supplement: Supplementary file 2 [file ijc0133-0346-SD2.doc]

**Supplementary Table 2.**

Ultra-deep pyrosequencing with sequencing reaction sizes of 119 bp, 168 bp, and 323 bp in patients without (#7, #11, #15, and #16), and with biphasic plasma-DNA distribution (#6, #10, #25, #38). For each sample, the top line indicates the percentage of mutated DNA fragments (column “% mutated”) and the number of reads (column “reads”), whereas the bottom line indicates the same after whole-genome amplification (WGA). The column labeled “Mutation” lists the *KRAS* mutation found in the respective primary tumor.

| **Patient** | **Mutation** | **119 bp** | | **168 bp** | | **323 bp** | |
| --- | --- | --- | --- | --- | --- | --- | --- |
|  |  | **% mutated** | **reads** | **% mutated** | **reads** | **% mutated** | **reads** |
| #7 | G13D (c.38G>A) | 0 | 11249 | 0 | 22059 | 0 | 18643 |
|  |  | 0 | 9939 | 0 | 22059 | 0 | 0 |
| #11 | G12V (c.35G>T) | 1.18 | 8082 | 0.31 | 9717 | 0 | 21010 |
|  |  | 0 | 7845 | 0 | 8498 | 0 | 3342 |
| #15 | G12D (c.35G>A) | 0 | 12649 | 6.9 | 11254 | 0 | 0 |
|  |  | 0 | 13401 | 0 | 22527 | 0 | 29546 |
| #16 | G12D (c.35G>A) | 0 | 9406 | 0 | 8553 | 0 | 6961 |
|  |  | 0.62 | 13271 | 0 | 11745 | 0.36 | 6049 |
|  |  |  |  |  |  |  |  |
| #6 | G12V (c.35G>T) | 39.25 | 14114 | 42.72 | 8978 | 48.34 | 6492 |
|  |  | 23.42 | 5479 | 9.61 | 12894 | 19.74 | 2989 |
| #10 | G12D (c.35G>A) | 33.06 | 189540 | 33.33 | 17608 | 27.24 | 22444 |
|  |  | 49.08 | 12270 | 28.42 | 21641 | 25.47 | 14447 |
| #25 | G12D (c.35G>A) | 17.11 | 13171 | 12.54 | 12163 | 0 | 4169 |
|  |  | 13.23 | 12732 | 8.59 | 13956 | 0 | 10537 |
| #38 | G12D (c.35G>A) | 37.47 | 11392 | 33.32 | 12778 | 27.37 | 21057 |
|  |  | 10.56 | 14596 | 4.56 | 14292 | 3.88 | 20450 |
