# Supplementary material for: Establishment of tumor-specific copy number alterations from plasma DNA of patients with cancer
Source: Int J Cancer. 2013 Jan 15;133(2):346–56. doi: 10.1002/ijc.28030 (PMC3708119; doi:10.1002/ijc.28030)
Supplement: Supplementary file 3 [file ijc0133-0346-SD3.doc]

**Supplementary Table 3.**

Sites of metastases and their association with the biphasic plasma DNA (bi-pDNA) distribution and number of CTCs (NA: not available).

|  | | | **Number of CTCs** | | | | | |
| --- | --- | --- | --- | --- | --- | --- | --- | --- |
| **Metastasis site** | **number of patients with bi-pDNA** | **number of patients without bi-pDNA** | **0** | **1** | **2-5** | **6-10** | **>10** | **NA** |
| bone | 4 | 2 | 1 |  |  |  | 4 | 1 |
| liver | 11 | 14 | 7 | 3 | 4 | 3 | 6 | 2 |
| lung | 1 | 10 | 4 | 3 | 2 |  | 1 | 1 |
| peritoneal carcinomatosis | 3 | 5 |  | 2 | 3 |  | 3 |  |
