# Supplementary material for: Establishment of tumor-specific copy number alterations from plasma DNA of patients with cancer
Source: Int J Cancer. 2013 Jan 15;133(2):346–56. doi: 10.1002/ijc.28030 (PMC3708119; doi:10.1002/ijc.28030)
Supplement: Supplementary file 4 [file ijc0133-0346-SD4.doc]

**Supplementary Table 4.**

Mapping of regions involved in artifacts after whole-genome amplification (WGA) of severely fragmented plasma-DNA. The first column lists the chromosomal region, the second and third columns the exact borders of the respective region, the fourth column the size of the region (in bp), the fifth column the number of oligos on the array-platform involved in the artifact and the column labeled “in/de” whether the artifact resulted in increased or decreased ratio values.

| **Chromosome** | **Start** | **End** | **Size (bp)** | **# Oligos** | **in/de** |
| --- | --- | --- | --- | --- | --- |
| 1p36.33-1p35.1 | 1:749625 | 1:33954225 | 33204600 | 810 | in |
| 1p34.3 | 1:36183989 | 1:38235282 | 2051293 | 54 | in |
| 1q21.2-1q22 | 1:149245378 | 1:155042676 | 5797298 | 262 | in |
| 2q24.2-2q31.1 | 2:162544755 | 2:170471439 | 7926684 | 174 | de |
| 2q31.1-2q32.3 | 2:182580451 | 2:196295211 | 13714760 | 204 | de |
| 2q37.3 | 2:240714768 | 2:242690037 | 1975269 | 50 | in |
| 4p16.3 | 4:950001 | 4:3550117 | 2600116 | 54 | in |
| 4q13.1-4q13.3 | 4:65962632 | 4:74827416 | 8864784 | 149 | de |
| 4q32.1 | 4:156021483 | 4:157480844 | 1459361 | 34 | de |
| 4q32.3-4q34.1 | 4:167206304 | 4:175832122 | 8625818 | 164 | de |
| 5q12.1-5q12.3 | 5:60977290 | 5:65345244 | 4367954 | 79 | de |
| 6p21.33-6p21.31 | 6:30422708 | 6:34897675 | 4474967 | 199 | in |
| 6p21.2 | 6:36759419 | 6:38701549 | 1942130 | 59 | in |
| 6p21.1 | 6:41227347 | 6:43038254 | 1810907 | 56 | in |
| 6q21-6q22.33 | 6:114397770 | 6:129094132 | 14696362 | 254 | de |
| 8q13.3-8q21.12 | 8:71939727 | 8:79170796 | 7231069 | 109 | de |
| 9q33.3-9q34.3 | 9:128026884 | 9:140128743 | 12101859 | 358 | in |
| 11p15.5 | 11:1 | 11:2246648 | 2246648 | 49 | in |
| 11q12.2-11q13.4 | 11:60699074 | 11:72872679 | 12173605 | 439 | in |
| 12q21.31-12q22 | 12:81394905 | 12:94217898 | 12822993 | 179 | de |
| 12q22-12q23.1 | 12:95315313 | 12:96618701 | 1303388 | 29 | de |
| 12q24.23-12q24.31 | 12:119017413 | 12:124390885 | 5373472 | 174 | in |
| 12q24.33 | 12:130299366 | 12:132231339 | 1931973 | 59 | in |
| 13q14.3-13q21.33 | 13:52406229 | 13:72583724 | 20177495 | 279 | de |
| 13q22.3-13q32.1 | 13:78632731 | 13:96195296 | 17562565 | 279 | de |
| 14q32.32-14q32.22 | 14:103527487 | 14:105467584 | 1940097 | 57 | in |
| 16p13.3 | 16:1 | 16:5856947 | 5856947 | 199 | in |
| 16p12.2-16p11.2 | 16:22221294 | 16:31412133 | 9190839 | 249 | in |
| 16q21-16q22.1 | 16:65151089 | 16:67433141 | 2282052 | 124 | in |
| 16q22.1-16q22.3 | 16:68534348 | 16:73524277 | 4989929 | 124 | in |
| 16q23.3-16q24.2 | 16:81990225 | 16:88610804 | 6620579 | 209 | in |
| 17p13.3-17p13.1 | 17:1 | 17:8970183 | 8970183 | 334 | in |
| 17p11.2-17q11.1 | 17:16186659 | 17:25018087 | 8831428 | 244 | in |
| 17q11.2 | 17:26473801 | 17:28216578 | 1742777 | 49 | in |
| 17q12-17q21.32 | 17:33805689 | 17:45980825 | 12175136 | 494 | in |
| 17q24.3-17q25.3 | 17:69712217 | 17:78623226 | 8911009 | 319 | in |
| 19p13.3-19p13.11 | 19:1 | 19:19854958 | 19854958 | 844 | in |
| 19q13.12-19q13.2 | 19:37823098 | 19:41577039 | 3753941 | 159 | in |
| 19q13.2-19q13.43 | 19:42864333 | 19:60859667 | 17995334 | 799 | in |
| 19q13.43 | 19:63251545 | 19:63784386 | 532841 | 34 | in |
| 20p13 | 20:1 | 20:3012700 | 3012700 | 74 | in |
| 20p11.21-20q11.23 | 20:25213567 | 20:36325723 | 11112156 | 224 | in |
| 20q13.11-20q13.2 | 20:41756635 | 20:49881938 | 8125303 | 224 | in |
| 20q13.31-20q13.32 | 20:55135683 | 20:57033197 | 1897514 | 49 | in |
| 20q13.33 | 20:59271072 | 20:62363629 | 3092557 | 107 | in |
| 21q11.2-21q21.1 | 21:14847723 | 21:17047504 | 2199781 | 49 | de |
| 21q22.2-21q22.3 | 21:42395372 | 21:46892353 | 4496981 | 169 | in |
| 22q11.1-22q11.23 | 22:15476913 | 22:23931833 | 8454920 | 219 | in |
| 22q12.1-22q13.33 | 22:27437979 | 22:49524240 | 22086261 | 629 | in |
