# Supplementary figures and images for: Establishment of tumor-specific copy number alterations from plasma DNA of patients with cancer
Source: Int J Cancer. 2013 Jan 15;133(2):346–56. doi: 10.1002/ijc.28030 (PMC3708119; doi:10.1002/ijc.28030)

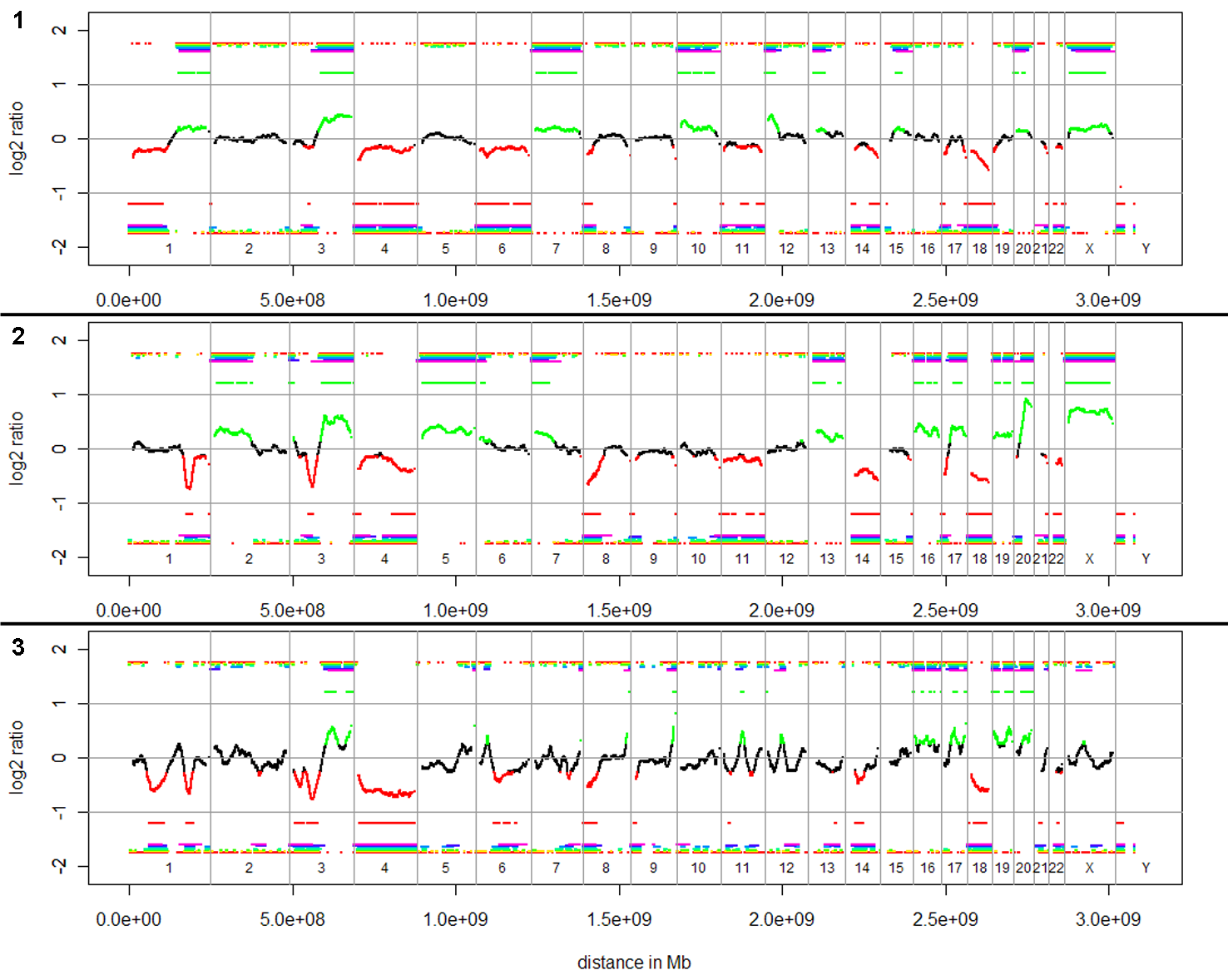

Supplement: Supplementary file 5 [file ijc0133-0346-SD5.tif]

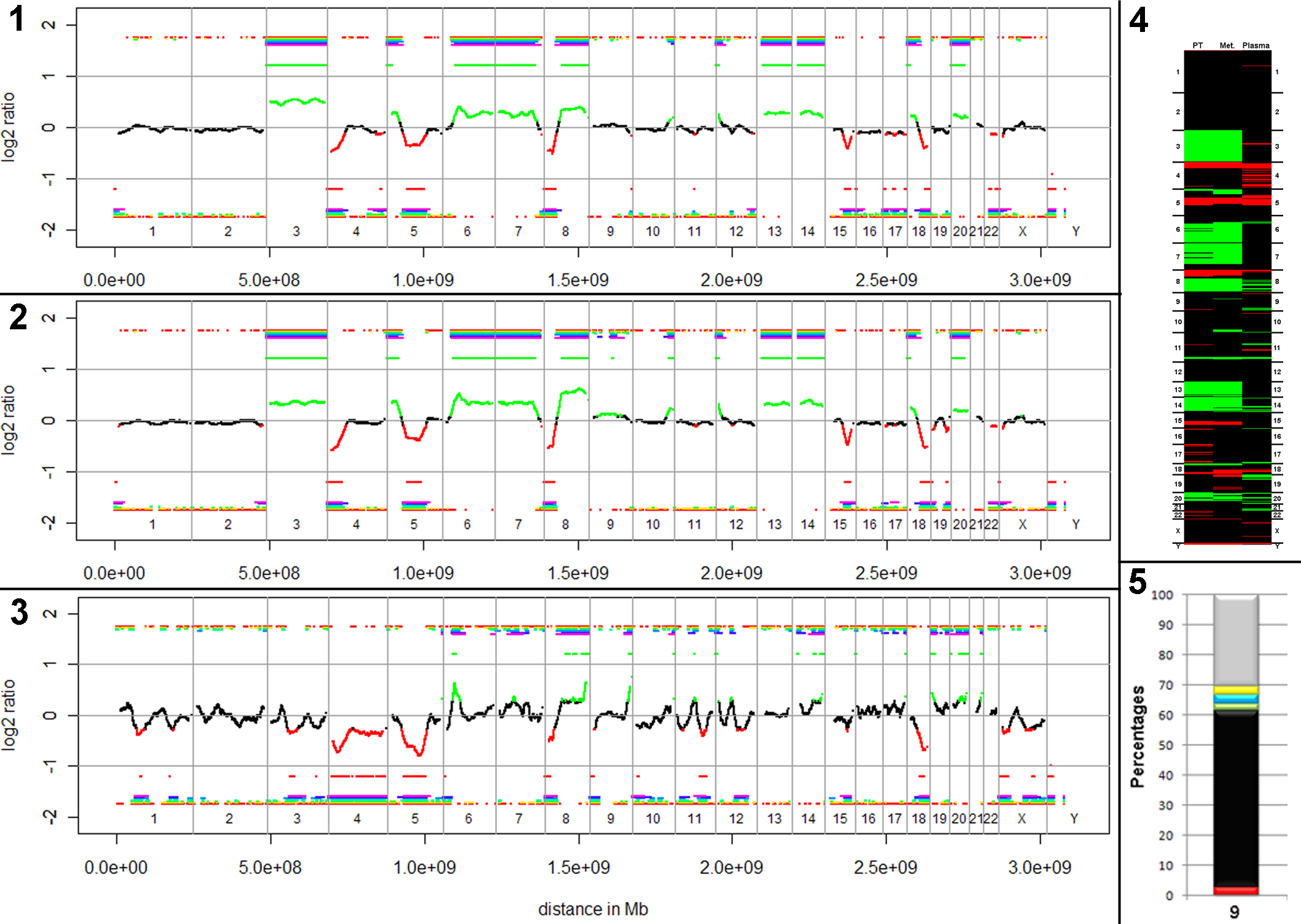

Supplement: Supplementary file 6 [file ijc0133-0346-SD6.tif]

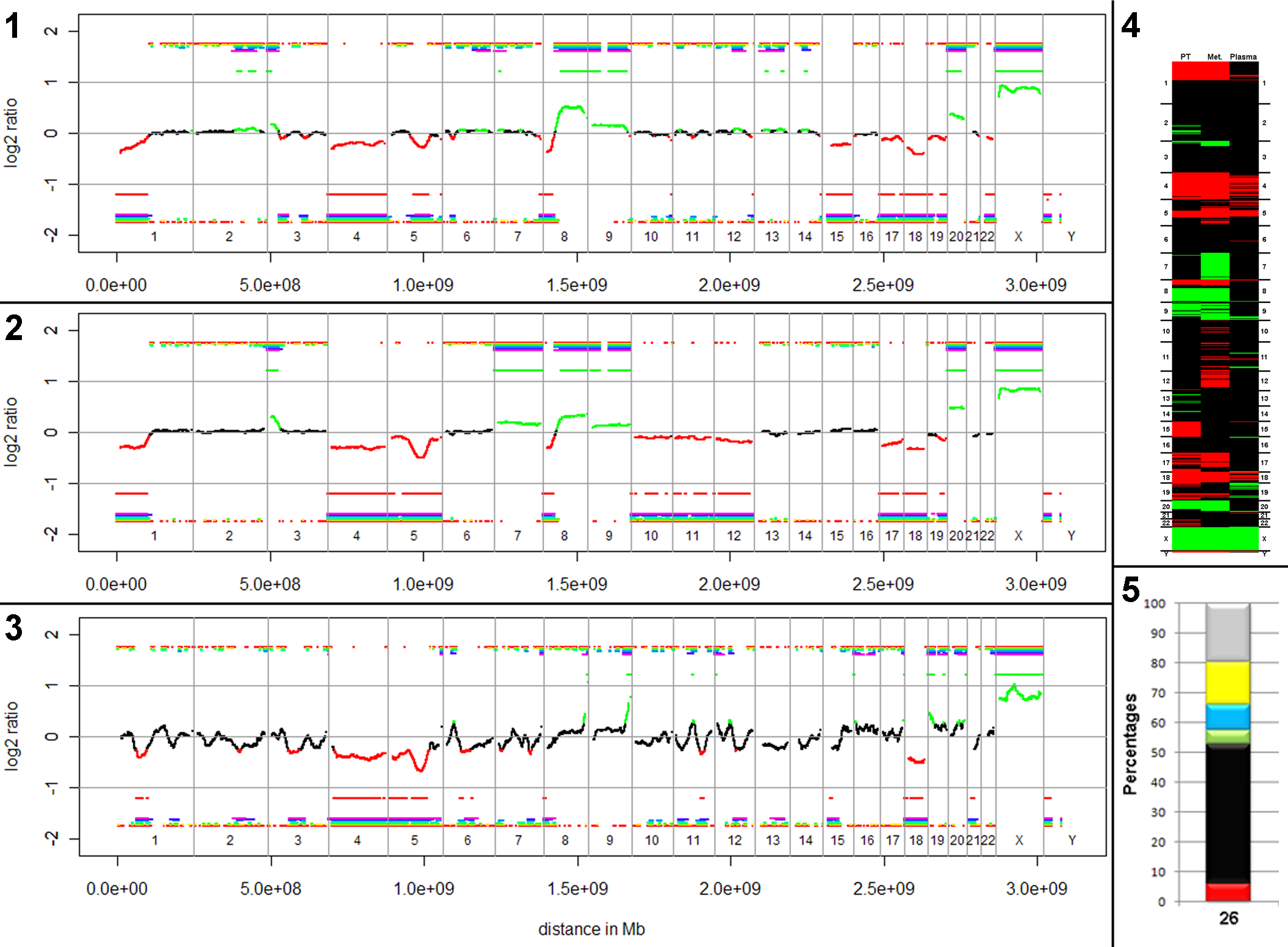

Supplement: Supplementary file 7 [file ijc0133-0346-SD7.tif]

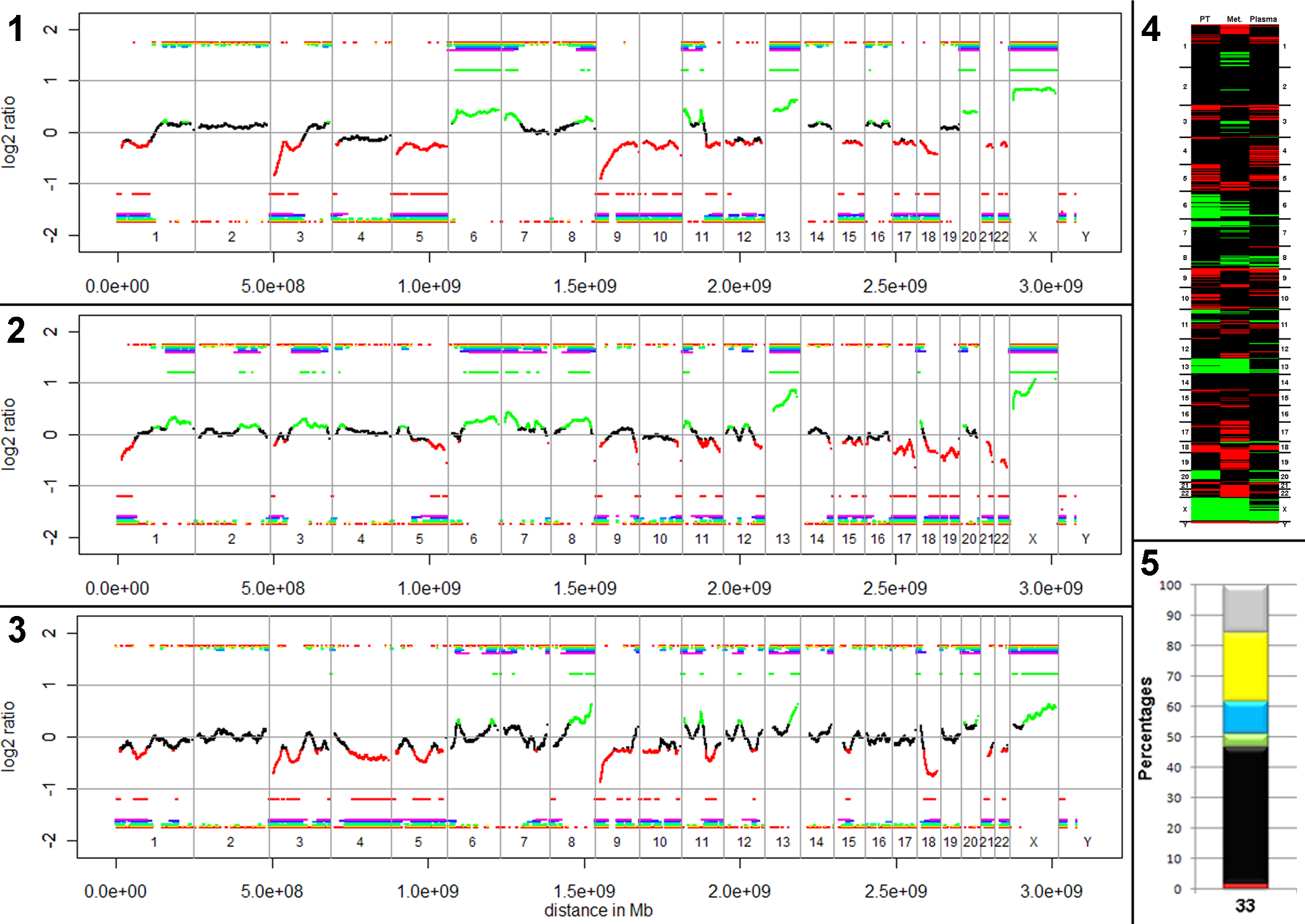

Supplement: Supplementary file 8 [file ijc0133-0346-SD8.tif]

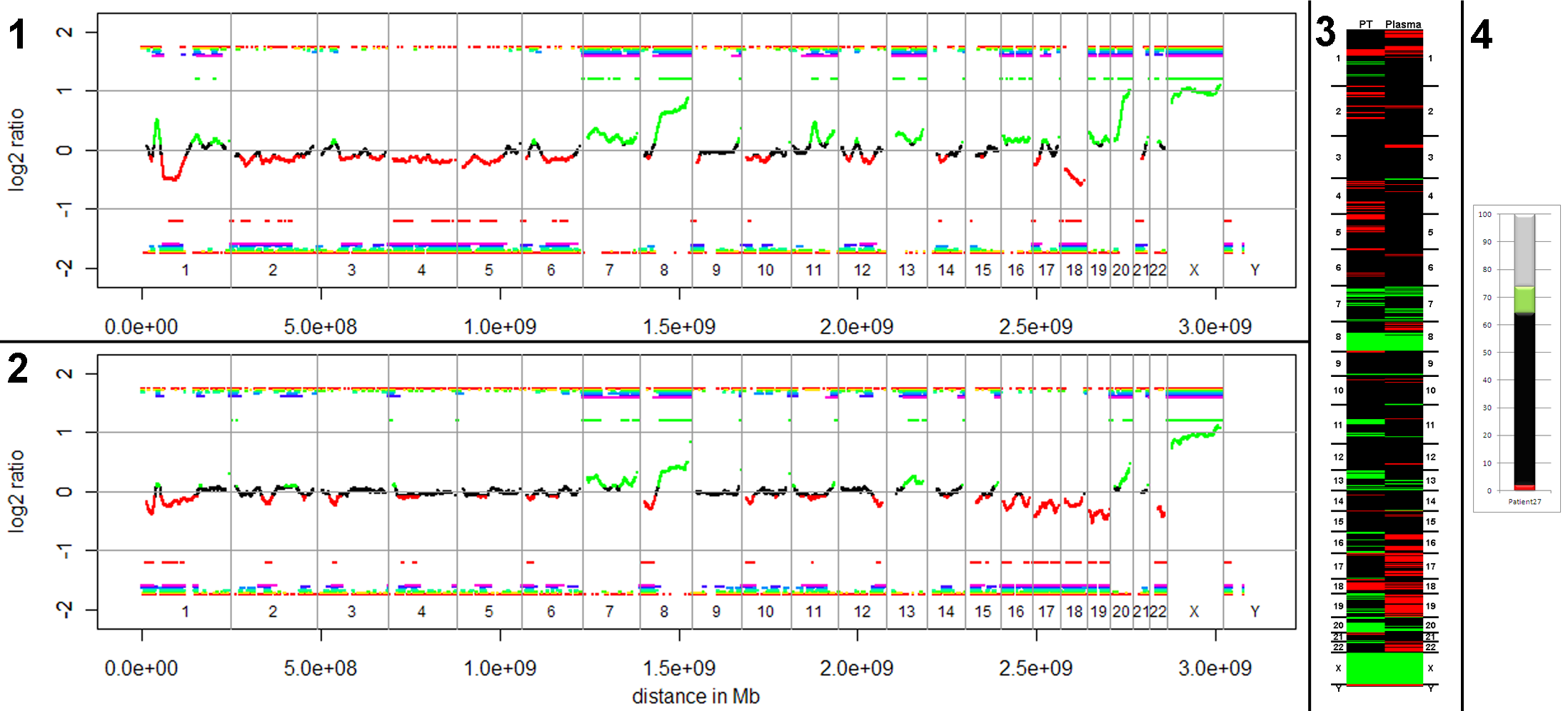

Supplement: Supplementary file 9 [file ijc0133-0346-SD9.tif]

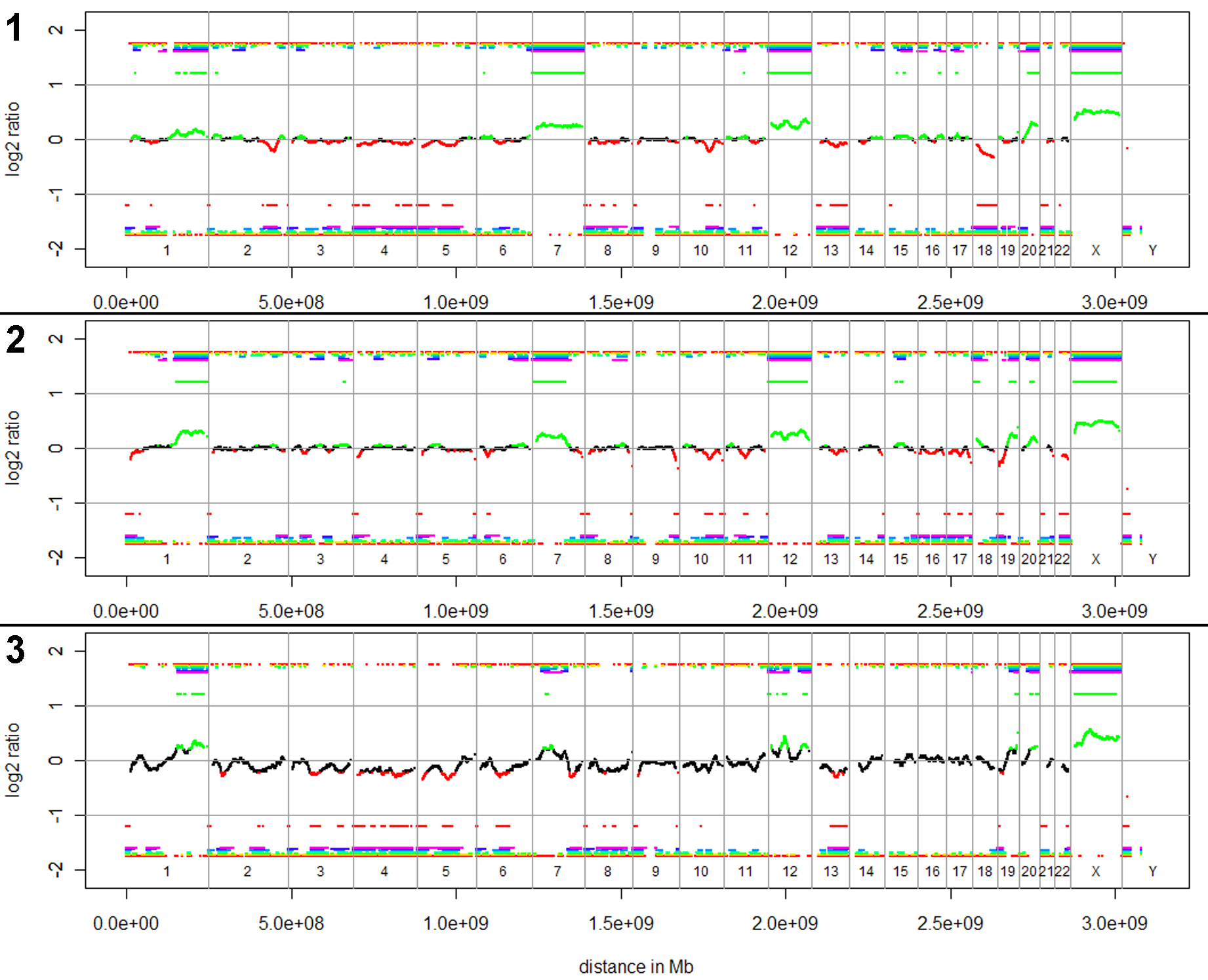

Supplement: Supplementary file 10 [file ijc0133-0346-SD10.tif]

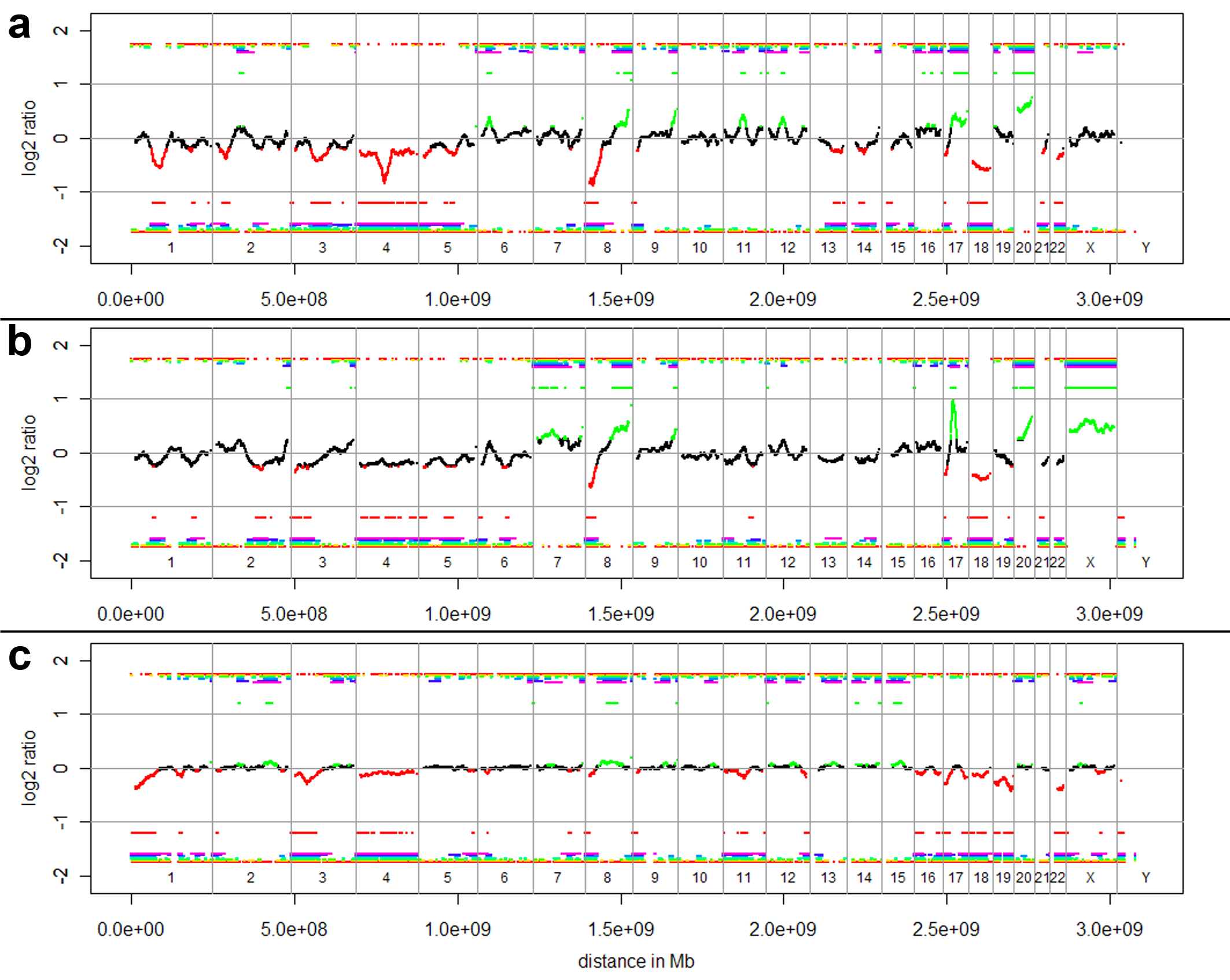

Supplement: Supplementary file 11 [file ijc0133-0346-SD11.tif]

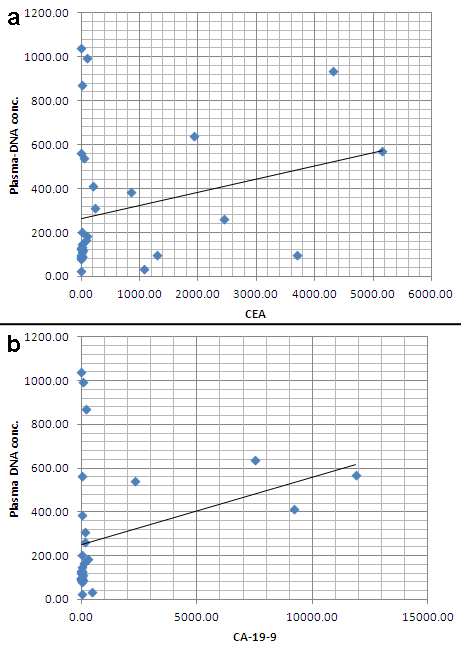

Supplement: Supplementary file 12 [file ijc0133-0346-SD12.tif]

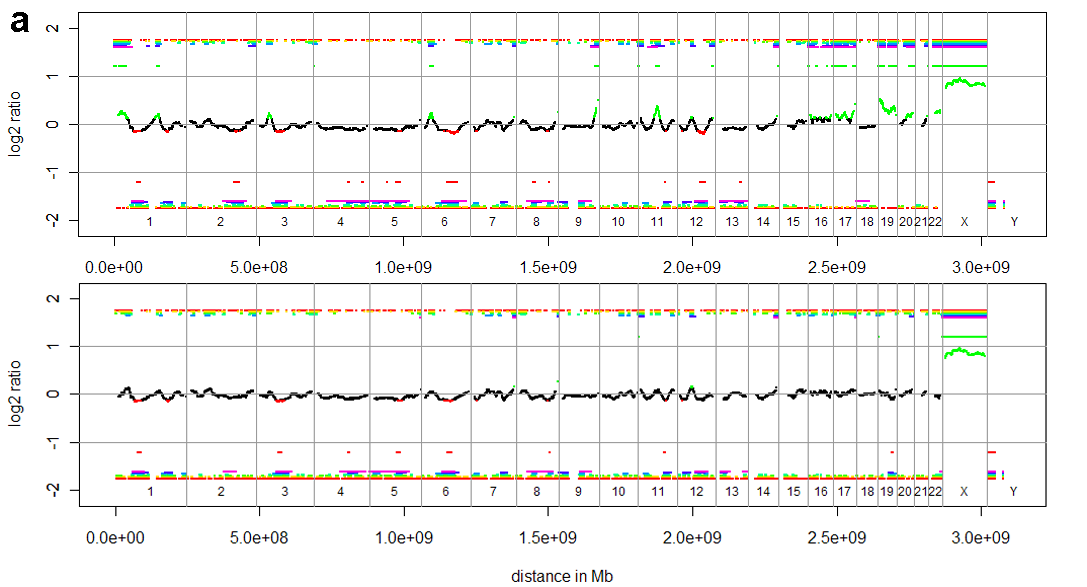

Supplement: Supplementary file 13 [file ijc0133-0346-SD13.tif]

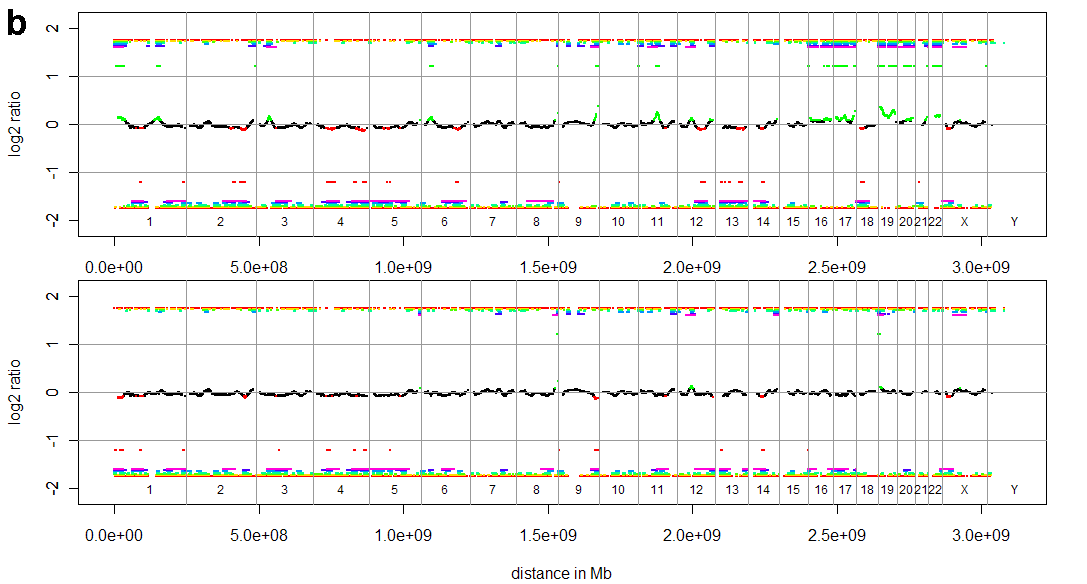

Supplement: Supplementary file 14 [file ijc0133-0346-SD14.tif]
