# Supplementary material for: Establishment of tumor-specific copy number alterations from plasma DNA of patients with cancer
Source: Int J Cancer. 2013 Jan 15;133(2):346–56. doi: 10.1002/ijc.28030 (PMC3708119; doi:10.1002/ijc.28030)
Supplement: Supplementary file 15 [file ijc0133-0346-SD15.docx]

**Supplementary Clinical Information**

*Patients*

All patients with colon cancer (n=32) had advanced stage (UICC Stage IV), progressive disease at the time of blood collection. Clinical evidence of disease progression was based on (a) newly diagnosed metastatic disease, (b) disease progression after initial treatment response during treatment break, or (c) primary tumor progression as initial response to anti-tumor treatment. Twenty-three patients were male, 9 female, and the mean age at diagnoses was 68 years (range: 45-81 years) (Supp. Table 1).

The intervals between diagnosis of the primary tumor and our blood collection, between diagnosis of the primary tumor and metastasis, and between blood collection and last therapy are listed in Supp. Table 1. The interval between diagnosis of the primary tumor and blood collection to obtain both plasma DNA and circulating tumor cells (CTCs) varied extensively within a range from 1 month (patients #2, #9, #18, #23) to more than 12 years (151 months; patient #28) (mean: 32 months; median: 24 months) (Supp. Table 1). The mean interval between blood collection and last therapy for all patients together was 104 days (median: 47 days; range: 0-688 days).

In addition to the clinical data in the main text we provide here some data regarding patients #9, #33, and #38:

Patient #9: At the time of diagnosis metastases were present in liver, bone, and abdomen (peritoneal carcinomatosis). We obtained biopsies from the primary tumor and the metastasis and performed our analyses from the peripheral blood one month after diagnosis.

Patient #33: At the time of diagnosis metastases were present in liver and abdomen (peritoneal carcinomatosis and abdominal wall metastasis). The primary tumor was completely removed and a liver biopsy was obtained four months later; our analyses were done 9 months after initial diagnosis.

Patient #38 had the primary tumor completely resected at diagnosis. Fourty-four months later metastases were noted in liver, bone, and lung; a biopsy was obtained from the liver. One month later we performed our analyses.

The most frequent sites of metastases were liver (diagnosed in 25 patients), followed by lung (*n*=11), peritoneal carcinomatosis (*n*=8) and bone (*n*=6). Supp. Table 3 summarizes how often we identified the biphasic plasma DNA size distribution in these patients and how many CTCs were found.

In patients #6 and #9 the primary tumor was only biopsied, in patients #26, #33, and #38 the primary tumor was resected completely. In three of these patients (i.e. #6, #9, #33) metastases were already present at the time of diagnosis of the primary tumor, in the remaining patients (i.e. #26 and #38) the interval between diagnosis of the primary tumor and metastasis was 31 and 44 months, respectively. At the time of our blood collection all of these patients had metastases at several different sites (Supp. Table 1).

The study was approved by the local ethics committee and written informed consent was obtained from all patients. The colon cancer patients were seen at the Division of Clinical Oncology, Department of Internal Medicine, at the Medical University of Graz. The blood from patients with repeated blood drawings on 5 consecutive days was obtained from the LKH Leoben. The breast cancer patients were treated at the Department of Obstetrics and Gynecology, Medical University of Graz.

**Supplementary Material and Methods**

*Quantitative analysis of plasma DNA*

For quantification of cell-free DNA in plasma we used Quant-iT™ PicoGreen ® dsDNA Kit (Invitrogen, Carlsbad, CA, USA) according to the manufacturer’s instructions. We used bacteriophage lambda DNA for a standard curve (ranging from 500 pg/µl to 16 pg/µl). 50 µl of the standard dilutions were transferred into disposable cuvettes and mixed with 50 µl of Quant-iT™ PicoGreen® reagent working solution. Experimental DNA samples were diluted in 1xTE (1:25) to a final volume of 50 µl and also mixed with 50 µl of Quant-iT™ PicoGreen® reagent working solution in disposable cuvettes. Fluorescence of the samples was then measured in duplicates using a QuantiFluor^TM^ Fluorometer (Promega, Madison, WI, USA). The fluorometer was calibrated using the blank and the highest concentration of the standard dilutions. DNA concentration of the samples was determined from the standard curve generated in Excel. From these DNA amount estimates we used 800 pg for a qualitative bioanalyzer analysis and, if available, 50 ng for the whole-genome amplification.

*Qualitative analysis of plasma DNA*

The length of the native plasma DNA was determined by electrophoresis on an Agilent 2100 Bioanalyzer using the DNA series Agilent High Sensitivity DNA kit (Agilent Technologies, Santa Clara, CA, USA). The Agilent 2100 Expert software (version B.02.07 or higher) offers a smear analysis with an integrator allowing size adjustments of the smear region. The software automatically determines the average size (bp), size distribution in CV (%), concentration (pg/μl), % of total, and molarity (pmol/l) for each defined smear region.

*BEAMing (Beads, Emulsions, Amplification, and Magnetics) Assay*

BEAMing involves single-molecule PCRs on magnetic beads in water-in-oil emulsions, allowing the quantitative detection of somatic mutations with high sensitivity and selectivity [^1^](#_ENREF_1) [^2^](#_ENREF_2). Our BEAMing analyses were performed by Inostics GmbH (Hamburg, Germany).

*DNA isolation of tumor DNA from FFPE sections*

Formalin-fixed paraffin embedded (FFPE) tissue samples from primary tumors and, if available, from metastases were cut and mounted on a microscope slide. Hematoxylin and eosin stained slides were reviewed by experienced pathologists (C.L. and S.L.) and areas with a high tumor cell infiltration were macrodissected from parallel sections. In addition, DNA was isolated using the QIAamp DNA FFPE tissue kit (Qiagen, Hilden, Germany), following the manufacturer’s instructions. Quantification and quality of the extracted DNA were determined using the Nano-Drop Spectrometer ND-1000 (Peqlab Biotechnologie, Erlangen, Germany).

*Generation of random DNA libraries and amplification of cell-free tumor DNA*

We adjusted the PCR procedure to minimize any further degradation of DNA and to allow an unbiased amplification of all fragments irrespective of their size according to our previous experience [^3^](#_ENREF_3) [^4^](#_ENREF_4). For our purposes, i.e. unbiased amplification of plasma DNA with fragments of different lengths, it has turned out that the GenomePlex Complete Whole Genome Amplification Kit (WGA2, Sigma-Aldrich, Vienna, Austria) is - with some modifications - best suited. In brief, we omitted the fragmentation step and directly prepared libraries. Amplification was performed by adding 7.5 µl of 10x Amplification Master Mix, 47.5 µl of nuclease-free water and 5 µl WGA DNA Polymerase to 10µl of DNA. Samples were amplified using an initial denaturation of 95°C for 3 min, followed by 20 cycles, each consisting of a denaturation step at 94°C for 15 s and an annealing/extension step at 65°C for 5 min. After purification using the GenElute PCR Clean-up Kit (Sigma-Aldrich, Vienna, Austria), DNA concentration was determined by a Nanodrop spectrophotometer. We used 50 ng of plasma DNA as starting template for the whole-genome amplification, as suggested for fragmented DNA samples by the manufacturer. After amplification the mean concentration of DNA was 136.01 ng/µl (min 57.32 ng/µl; max 241.12 ng/µl).

*Array CGH*

Array CGH was carried out using a genome-wide oligonucleotide microarray platform (Human genome CGH 60K microarray kit, Agilent Technologies, Santa Clara, CA, USA), following the manufacturer’s instructions (protocol version 6.0). We used commercially available male reference DNA (Promega, Madison, WI, USA). DNA from FFPE samples and the CTCs, which were amplified with Phi 29 polymerase, were additionally digested with ALUI/RSAI. Samples were labeled with the Bioprime array CGH genomic labeling system (Invitrogen, Carlsbad, CA, USA) according to the manufacturer’s instructions. In brief, 500 ng test DNA and reference DNA were differentially labeled with dCTP-Cy5 or dCTP-Cy3 (GE Healthcare, Milwaukee, WI, USA). Slides were scanned using a microarray scanner, and images were preprocessed using Feature Extraction and DNA Workbench 5.0.14 (Agilent Technologies).

Evaluation of our array CGH was done based on our previously published algorithm [^3^](#_ENREF_3) [^4^](#_ENREF_4) in R [^5^](#_ENREF_5). In brief, data normalization and calculation of ratio values were conducted employing the Feature Extraction software 9.1 from Agilent Technologies. The algorithm focuses on detecting which ratio values differ significantly from the ratio profile’s mean. The algorithm includes the employment of running means with different window sizes and analyses at progressively greater levels of smoothing and subsequently combining of these analyses. Consecutive data points are combined and their mean ratio values are presented in graphs. The algorithm iterates through the profile by changing window positions, employing a sliding window approach, followed by calculations of the mean ratio value for each window based on the respective ratio values. Assuming that a window’s ratio values are distributed normally, we estimate the standard deviation (SD) by considering the outmost value that is within ± 34.1% of the mean. Due to the noise with plasma DNA thresholds were defined stringently as ± 1.5 times the SD. The values obtained were assigned to all oligos of the respective window. We used 7 different window sizes consisting of 10, 25, 50, 100, 250, 500, and 750 adjacent oligos. Depending on the window size they will be labeled with a different color and distance to the X-axis, thus generating a color bar code. As we use sliding windows the assessment of the copy number status of each oligo is based on 44 different calculations. The final assessment is indicated in a single green or red bar for gained or lost regions, respectively, which is generated if at least 39 (90%) of the 44 repetitive calculations consistently result in the same copy number change. Furthermore, the algorithm generates a table with all localizations of significant calls which allows detailed mapping of each CNV. All ratio profiles shown in the center of the images were calculated using a 500 oligonucleotide window.

*Plasma DNA array CGH artifact correction*

We generated random DNA libraries by converting the plasma DNA fragments to PCR-amplifiable OmniPlex Library molecules flanked by universal priming sites for whole genome amplification (WGA). We subjected the WGA products to array CGH on a 60K microarray platform.

Copy number estimations after WGA may have to deal with potential amplification artifacts, which may result in under- (e.g. allele drop out) or over-representations (e.g. preferential amplifications). Thus, a systematic amplification bias had not been observed with the single cell amplification products as we had previously reported [^3^](#_ENREF_3) [^4^](#_ENREF_4), we had not employed corrections for single cell analyses. However, with the plasma DNA WGA products we noted amplification biases and, because a large part of this amplification bias was systematic (e.g. correlated with GC-content of the DNA), we were able to account for this bias as follows. Firstly, we mapped the systematic amplification biases in healthy controls (3 females and 3 males) (Supp. Table 4) (illustrated in Supp. Fig. 9a-b). We then introduced corrections for these regions in our algorithm for plasma DNA. In brief, for the regions listed in Supp. Table 4 we increased the number of repetitive calculations, which had to result consistently in the same copy number change from 39 (90%) of the 44 calculations to 41 (93%), requiring that the copy number change is also indicated at least in the 500 or 750 window size calculation. For the representation of the ratio profile in the center of our array CGH illustrations these regions were adjusted with a correction factor, which dependeds on the aforementioned calculations. Female and male array CGH healthy control profiles before and after these corrections are shown in Supp. Fig. 9. The same corrections were applied in all plasma DNA analyses of the tumor patients.

*Deep sequencing with the 454 GS FLX genome sequencer*

“Picotiter plate pyrosequencing”, a massively parallel sequencing-by-synthesis approach, relies on emulsion PCR-based clonal amplification of a DNA library adapted to micron-sized beads and subsequent pyrosequencing-by-synthesis of each clonally amplified template in a picotiter plate, generating up to 1.000.000 unique clonal sequencing reads per experiment [^6^](#_ENREF_6). Sequence variants that represent a fraction of a complex sample can be vastly oversampled, thus enabling statistically meaningful quantification of low-abundance variants.

For amplicon preparation of *KRAS* we used a proof-reading enzyme (Fast Start High Fidelity System, Roche Diagnostics) according to the manufacturer’s instructions with 35 cycles of amplification. Primers were designed resulting in 3 different amplicon lengths (*KRAS* specific length 119 bp, 168 bp, and 323 bp), including the Roche-compatible adaptors A and B with a length of 21 bp, plus 4 bp TCAG key sequence (each read has to start with key sequence) and 10 bp MID (multiple identifier; barcode). Amplicons from patient and control samples (cell-free native and whole genome amplified DNA from plasma) were purified, quantified with PicoGreen, and pooled equimolarily (6-8 samples per pool). Amplicon pools were quality checked on an Agilent Bioanalyzer using Agilent High Sensitivity DNA kit. Emulsion PCR was performed according to emPCR Method Manual – Lib-A MV. In doing so, two emulsion PCRs were performed for bidirectional sequencing (forward and reverse). Microbeads were enriched and deposited onto PicoTiterPlates provided for the 454-FLX instrument (Roche Diagnostics). Massively parallel pyrosequencing was performed according to the manufacturer's protocol. Base calls and quality scores were generated using the GS Run Processor software on a HPC-cluster, and variants were extracted using the GS Amplicon Variant Analysis 2.6 software provided with the platform. Read lengths strongly corresponded to amplicon lengths. The average coverage was 57045 x for the 11 9bp fragment, 12201 x for the 168 bp fragment, and 13540 x for the 323 bp fragment, respectively. Deep sequencing with fragments even longer than 323 bp is challenging because the number of sequencing reads decreases. For the 470 bp fragment length, the number of reads was too low for unequivocal interpretations. The DNA preparations were sequenced with a GS FLX genome sequencer (454 LifeSciences/Roche Diagnostics) according to the manufacturer’s instructions.

*Deep sequencing of DNA fragments after whole-genome amplification*

We subjected the random DNA libraries generated during the WGA process (from the same patients as above) to deep sequencing to estimate whether shifts between the ratios of mutated versus non-mutated DNA fragments had occurred during the amplification process. We did not detect *KRAS* mutations in the WGA-products of the four patients (#7, #11, #15, #16) with low frequency or absent *KRAS* mutation in their native DNA (Supp. Table 2). For patients with a high frequency of *KRAS* mutation (#6, #10, #15, #38) the percentage of mutated DNA fragments was about the same as in the native DNA in patients #10 and #25; however, this percentage was lower in patient #6 and even notably lower in patient #38 (Supp. Table 2). This suggests that WGA may cause a shift towards a higher number of non-mutated DNA fragments in a subset of cases.

*Statistics*

Statistical analysis was done using Microsoft Excel or R for the Wilcoxon Rank Sum test, which was used for the comparisons of plasma DNA concentrations and CTCs of patients with and without biphasic plasma DNA size distributions. Unpaired two-sided Student’s t-tests were used to calculate *P* values for the other data sets.

**Supplemental References**

1. Diehl F, Li M, Dressman D, He Y, Shen D, Szabo S, et al. Detection and quantification of mutations in the plasma of patients with colorectal tumors. *Proceedings of the National Academy of Sciences of the United States of America* 2005;102(45):16368-73.

2. Diehl F, Li M, He Y, Kinzler KW, Vogelstein B, Dressman D. BEAMing: single-molecule PCR on microparticles in water-in-oil emulsions. *Nature methods* 2006;3(7):551-9.

3. Geigl JB, Obenauf AC, Waldispuehl-Geigl J, Hoffmann EM, Auer M, Hormann M, et al. Identification of small gains and losses in single cells after whole genome amplification on tiling oligo arrays. *Nucleic acids research* 2009;37(15):e105.

4. Geigl JB, Speicher MR. Single-cell isolation from cell suspensions and whole genome amplification from single cells to provide templates for CGH analysis. *Nature protocols* 2007;2(12):3173-84.

5. Team RDC. R: A language and environment for statistical computing.: R Foundation for Statistical Computing, Vienna, Austria. , 2009.

6. Margulies M, Egholm M, Altman WE, Attiya S, Bader JS, Bemben LA, et al. Genome sequencing in microfabricated high-density picolitre reactors. *Nature* 2005;437(7057):376-80.

**Supplementary Figures**

**Supplementary Figure 1**

Evaluation of available material for patient #6.

For all array CGH profiles the multicolor bar codes at the top or bottom of the ratio profiles illustrate the results obtained during the iterative calculations with various window sizes, the single green and red bars summarize the regions which were gained or lost based on all calculations (Methods). Black parts in the profile represent balanced regions, lost regions appear in red and gained regions in green. The respective heat maps show the copy number status (red: under-represented; black: balanced; green: over-represented). The bar charts display the percentages of chromosomal regions, which were commonly lost (red), balanced (black), or gained (green) in all analyzed samples; shared by metastasis and plasma DNA only (blue); shared by primary tumor and plasma DNA only (yellow); or unique to the plasma DNA (gray).

(a) Array CGH profiles of the primary tumor (panel 1), metastasis (panel 2), and plasma DNA (panel 3) for patient #6.

**Supplementary Figure 2**

Analysis of material from patient #9.

Array CGH profiles of the primary tumor (panel 1), metastasis (peritoneal carcinomatosis; panel 2), plasma DNA (panel 3), heat map (panel 4), and bar chart (panel 5). For explanations regarding the color codes see legend of Supplemetary Figure 1.

**Supplementary Figure 3**

Analysis of material from patient #26.

Array CGH profiles of the primary tumor (panel 1), liver metastasis (panel 2), plasma DNA (panel 3), heat map (panel 4), and bar chart (panel 5). For explanations regarding the color codes see legend of Supplemetary Figure 1.

**Supplementary Figure 4**

Analysis of material from patient #33.

Array CGH profiles of the primary tumor (panel 1), metastasis (liver; panel 2), plasma DNA (panel 3), heat map (panel 4), and bar chart (panel 5). For explanations regarding the color codes see legend of Supplemetary Figure 1.

**Supplementary Figure 5**

Analysis of material from patient #27.

Analysis of primary tumor (panel 1), plasma DNA (panel 2), and heat map (panel 3). The bar chart (panel 4) indicates percentages of commonly lost (red), gained (green), and balanced (black) oligonucleotides. Gray indicates copy number changes observed only in the plasma DNA, but not in the primary tumor.

**Supplementary Figure 6**

Analysis of material from patient #38.

Array CGH profiles of the primary tumor (panel 1), liver metastasis (panel 2), and plasma DNA (panel 3).

**Supplementary Figure 7**

Plasma DNA profiles from three patients for which no further tumor material was available for analysis, i.e. patient #10 (a); patient #20 (b); patient #25 (c).

**Supplementary Figure 8**

Correlation with tumor markers:

(a) CEA (slope of the linear regression line: 0.0601; correlation coefficient: 0.27).

(b) CA19-9 (graph without patient 6, whose CA19-9 value was exceptional high with 370,448; Slope of the linear regression line without patient 6: 0.0308; correlation coefficient: 0.315; with patient 6: slope: 0.0018; correlation coefficient: 0.394).

**Supplementary Figure 9**

Array CGH analysis after whole genome amplification products from plasma DNA.

(a) Plasma DNA analysis from female healthy donor F3 showing artifacts as listed in Supplementary Table 6 before (upper panel) and after correction (lower panel). Note that the overrepresentation of the X-chromosome (male reference DNA) is clearly visible because male reference DNA was used.

(b) As in (a), but with plasma DNA from male healthy donor M2 before (upper panel) and after (lower panel) artifact correction.
